# Economic policy uncertainty and medical inflation in China: multi-scale linkages and time-varying responses

**DOI:** 10.3389/fpubh.2026.1910486

**Published:** 2026-10-14

**Authors:** Liyan Pang, Dawei Zhou, Yuhan Wang

**Affiliations:** 1 School of Artificial Intelligence and Computer Science, Jilin University of Finance and Economics, Changchun, Jilin, China; 2 School of Economics, Jilin University, Changchun, Jilin, China; 3 China-Japan Union Hospital of Jilin University, Changchun, Jilin, China

**Keywords:** economic policy uncertainty, health economics, health-care CPI, medical inflation, SV-TVP-FAVAR, wavelet analysis

## Abstract

**Introduction:**

Medical inflation is a central health-system cost pressure, yet little is known about whether economic policy uncertainty (EPU) affects medical prices differently across timescales and historical periods.

**Methods:**

This paper examines the multi-scale and time-varying relationship between EPU and medical inflation in China using quarterly data from 2004Q1 to 2026Q1, wavelet filtering, and an SV-TVP-FAVAR model. Posterior uncertainty is summarized by the posterior mean and the 16th and 84th percentiles.

**Results:**

Positive responses are present in both frequency-specific baseline models. The full-sample posterior-mean maxima for the higher- and lower-frequency EPU components are 0.297 and 0.108; they are 0.214 and 0.208 when the sample ends in 2025Q4. In the three-factor model, aggregate health-care CPI reaches 0.237 for a shock dated 2020Q2 at an eight-quarter horizon (central 68% credible interval [0.063, 0.412]). The eight-quarter posterior band overlaps those at several earlier dates, and the estimated magnitude varies with factor count. In the baseline full-sample specification, western medicine prices have the largest consumer-side posterior-mean maximum, reaching 0.424 for a shock dated 2020Q3 at six quarters (central 68% credible interval [0.231, 0.618]), although the exact peak date varies across specifications. Traditional Chinese medicine prices and medical service CPI show smaller positive responses, while pharmaceutical manufacturing PPI provides complementary evidence from the production side.

**Discussion:**

Overall, the results show that the EPU-medical inflation relationship varies across EPU frequency scales, historical dates, and health-price components.

## 1 Introduction

Medical inflation is a concrete health-system cost pressure, not merely a technical component of the consumer price index. When health-care prices rise, households may face greater affordability pressure, insurers and public payers face higher reimbursement burdens, and providers and pharmaceutical firms must adjust pricing, procurement, and production decisions. These consequences are especially important in a health system where public payment rules, pharmaceutical regulation, provider incentives, and household out-of-pocket burdens interact. For public health, the concern is not simply that an index increases: persistent medical price growth can weaken financial protection and place access to care under pressure when household income and health budgets do not keep pace. This paper examines economic policy uncertainty (EPU) as a macro-institutional source of that pressure. EPU refers to uncertainty about the direction, timing, and implementation of economic policy. The newspaper-based index developed by Baker et al. (1) provides a standard empirical measure because it captures policy-related uncertainty in public discussion rather than only realized market volatility or forecast disagreement. Although EPU has been linked extensively to macroeconomic activity and financial conditions, it remains unclear whether and how it enters China’s medical-price system.

The underlying problem is empirically unresolved because EPU can operate through competing mechanisms. Greater uncertainty may weaken spending and reduce price pressure through aggregate demand. In medical markets, however, it may also affect financing costs, input reliability, inventory decisions, procurement plans, reimbursement expectations, and provider pricing. These mechanisms do not necessarily operate at the same speed: policy news may be absorbed quickly, whereas contracts, production plans, reimbursement arrangements, and expectations may adjust over several quarters. Most EPU studies focus on real activity, financial markets, trade, or broad inflation. Health-economics and public-health research has examined medical prices, financial protection, hospital payment, insurance design, quality adjustment, and pharmaceutical policy, but seldom treats broad economic policy uncertainty as the organizing exposure. Consequently, the literature does not establish whether shorter- and longer-cycle movements in EPU are associated with different medical-price responses in China, whether these responses vary across historical episodes, or whether they are concentrated in particular parts of the health-price system.

Against this background, the study has three objectives. First, it determines whether medical inflation responds differently to shorter- and longer-cycle components of EPU. Second, it estimates how the response of aggregate medical inflation to an EPU shock changes across historical dates and quarterly horizons after broader macroeconomic conditions are taken into account. Third, it compares aggregate health-care CPI with traditional Chinese medicine prices, western medicine prices, medical service CPI, and pharmaceutical manufacturing PPI to identify where the response is most visible within the health-price system.

To pursue these objectives, the paper combines two linked tools rather than relying on a single average time-domain design. Wavelet filtering separates EPU into high- and low-frequency components, while medical inflation remains the unfiltered response variable. The SV-TVP-FAVAR model then traces the response of medical inflation to a one-standard-deviation EPU shock within a broader macroeconomic information set. Three latent factors summarize common macro-financial movements, and the response is allowed to vary over time instead of being held constant for the full sample. The quarterly sample covers China from 2004Q1 to 2026Q1. EPU is measured with the China index based on the South China Morning Post (SCMP), hosted by the Economic Policy Uncertainty project and constructed under the Baker et al. framework. Medical inflation is measured by the year-on-year growth rate of China’s health-care CPI. The analysis also considers traditional Chinese medicine prices, western medicine prices, medical service CPI, and pharmaceutical manufacturing PPI. Variables are transformed for stationarity through logarithms or differencing where appropriate. The final specification uses three principal-component factors from the standardized macroeconomic information set, and the Akaike information criterion selects a one-lag VAR. China is a useful setting because medical prices are jointly shaped by macroeconomic conditions and health-sector institutions, while the sample spans the global financial crisis, domestic policy adjustments, COVID-19, and the post-pandemic period.

The study is designed to contribute in three ways. It extends EPU analysis to medical inflation, a public-health-relevant price outcome connected to affordability and fiscal sustainability. It distinguishes price dynamics from expenditure growth, which also reflects utilization, insurance coverage, demographics, and public budget decisions. It also integrates a multi-scale analysis with a time-varying, factor-augmented model so that persistence, historical variation, and differences across consumer- and production-side health-price indicators can be examined within one framework. Together, these contributions link macroeconomic uncertainty to health-system affordability through medical-price dynamics.

## 2 Literature review and research gap

### 2.1 Measuring economic policy uncertainty and macroeconomic transmission

Uncertainty has been measured in several ways, with the choice of proxy generally following the mechanism under study. Existing measures include forecast errors, disagreement among forecasters, business surveys, financial-market volatility, and latent uncertainty factors extracted from large macroeconomic datasets (2–6). These measures are well suited to questions about investment, hiring, credit conditions, and real activity when uncertainty reflects the broader economic environment. They are less direct when policy itself is the source of uncertainty. For that purpose, the newspaper-based framework of Baker et al. (1) identifies EPU from articles that jointly discuss the economy, policy, and uncertainty. Related evidence on fiscal and monetary policy uncertainty further shows that policy-specific uncertainty can be transmitted to output, credit spreads, and wider macroeconomic activity (7, 8).

Within this measurement literature, the China setting raises an additional question of institutional fit. Policy communication, media coverage, and institutional arrangements differ from those in advanced economies, so a China measure cannot simply be assumed to reproduce U. S. or European information conditions. Huang and Luk (9) address this issue by constructing an EPU index from local newspapers and China-specific search terms. The present paper uses the SCMP-based China index distributed by the Economic Policy Uncertainty project and constructed within the Baker et al. (1) framework. This choice keeps the measure within the news-based EPU tradition while applying it to a health-price outcome rather than the more familiar outcomes of investment, output, and trade.

### 2.2 Uncertainty, inflation, and price dynamics

Turning from measurement to price consequences, the literature does not imply a single expected sign for the response of inflation to uncertainty. If uncertainty causes firms to delay investment and households to postpone spending, aggregate demand weakens and price pressure may fall. Leduc and Liu (10) provide a clear example: in their framework, an uncertainty shock resembles an adverse aggregate-demand shock, raising unemployment while lowering inflation. For the present study, this result cautions against assuming before estimation that an EPU shock must raise medical inflation.

That demand-side account is only part of the price-adjustment process. Past inflation instability, expectation formation, the anchoring of expectations, and uncertainty about future inflation shape how price responses evolve across regimes and over time (11–14). Health-sector institutions add another layer because reimbursement rules, procurement cycles, production planning, and expectations about public payment may slow or redirect cost pass-through. Accordingly, neither the sign nor the persistence of the medical-price response can be imposed in advance. Some effects may appear soon after policy news or shifts in market sentiment, whereas others may reach medical prices only after contracts, inventories, production plans, and payment expectations have adjusted.

### 2.3 Medical inflation as a distinct health-system price outcome

From a public-health perspective, the choice of outcome is consequential. Medical inflation is not equivalent to growth in health expenditure: expenditure combines prices with quantities, technology, insurance coverage, quality change, and public or private payment decisions. Medical price growth is narrower, but it is more directly connected to the price pressure faced by households and payers. Health-care CPI belongs to the consumer-price system, yet its movement is mediated by health-sector institutions and should not be treated as interchangeable with either broad health spending or ordinary consumer-price inflation. In this paper, medical inflation therefore refers to consumer-facing health-care price inflation measured by health-care CPI and its relevant components, with pharmaceutical manufacturing PPI used as complementary production-side evidence.

The measurement literature reinforces this distinction. Cutler et al. (15) and Dunn et al. (16) show that conventional medical price indices may misstate price growth when treatment quality, intensity, and product mix change. At the same time, health economics and industrial organization show that competition among providers and insurers, hospital-insurer bargaining, public reimbursement policy, and patient cost-sharing jointly shape prices, premiums, quantities, and spending (17–23). For example, insurer competition affects negotiated payment and premiums, hospital bargaining changes the prices received by providers, public payment rules can spill over into private physician fees, and cost-sharing can alter both utilization and the prices paid for care. The common implication is that medical prices are formed through payment systems, bargaining, regulation, technology, and quality change rather than in a frictionless market. This institutional structure also means that a macroeconomic disturbance may be absorbed differently across providers, insurers, pharmaceutical firms, and patients.

Beyond measurement, public-health scholarship places medical costs within the broader goals of financial protection, equitable access, and universal health coverage. Evidence from China shows that near-universal insurance coverage has not eliminated exposure to out-of-pocket costs or the need to strengthen financial protection, especially for vulnerable households (24). Reviews of China’s progress toward universal health coverage likewise identify gaps in financial protection, equity, service quality, and access to essential medicines as continuing health-system concerns (25). Hospital reform evidence is particularly relevant to the present outcome: changes in drug markups, service prices, and provider incentives can redistribute expenditure across medicines, diagnostic services, and levels of care rather than simply reduce the overall burden (26). These studies do not examine EPU, but they establish why medical price dynamics have public-health significance: price pressure can affect financial protection, access, and the sustainability of health financing even when insurance coverage is extensive.

This perspective also clarifies why expenditure and price evidence answer different public-health questions. An increase in expenditure can accompany improved access, expanded insurance benefits, greater service use, technological change, or a larger public budget; it is not necessarily evidence of worsening affordability. Conversely, broad insurance coverage does not by itself guarantee effective financial protection if benefit ceilings, deductibles, copayments, or uncovered medicines leave households exposed to rising prices (24, 25). Provider-payment and hospital-pricing reforms may further shift costs among drugs, medical services, diagnostic procedures, insurers, and patients, so the distribution of price changes within the health system matters as much as the aggregate spending total (26). Health-care CPI cannot measure every one of these burdens directly, but it offers a consistent consumer-facing indicator of price pressure. Reading it together with medicine-price components, medical service CPI, and pharmaceutical manufacturing PPI therefore provides a layered view of affordability and system financing that expenditure alone cannot supply.

These public-health concerns make China’s pharmaceutical pricing institutions especially relevant for component-level analysis. National centralized drug procurement and related reforms create a direct connection between policy design and medicine prices. Evaluations of the “4 + 7” pilot and subsequent centralized procurement document substantial changes in the prices of procured medicines and in related pharmaceutical price patterns (27, 28). Those studies do not identify the EPU channel examined here; instead, they show why medicine-price components are meaningful outcomes within China’s health-price system. The pharmaceutical manufacturing PPI analysis then extends the inquiry upstream by asking whether uncertainty-related price dynamics also appear in pharmaceutical production. Together, the consumer- and production-side measures connect macroeconomic uncertainty to a pricing environment faced by households, providers, firms, and public payers.

### 2.4 Multi-scale and time-varying methods

Methodologically, the empirical question requires attention to both timing and frequency. A full-sample correlation or a VAR with fixed coefficients can provide a useful benchmark, but it compresses short fluctuations, persistent movements, crisis episodes, and calmer periods into a single average relationship. Wavelet methods retain information about when a movement occurs and the frequency band to which it belongs. Ramsey and Lampart (29), Torrence and Compo (30), and Grinsted et al. (31) provide the foundations for time-frequency co-movement analysis, while Baqaee (32) shows how wavelets can isolate frequency-specific movements in inflation. Time-varying VAR methods address the other dimension of the problem. Primiceri (33) develops a structural VAR with changing parameters and stochastic volatility, Del Negro and Primiceri (34) refine the stochastic-volatility estimation procedure, and Bernanke et al. (35) introduce FAVAR to summarize a large macroeconomic dataset with a small set of factors. Combining these elements allows the model to retain broad macroeconomic information while tracing changes in EPU transmission over time.

The two methods therefore have distinct but complementary roles. Wavelet analysis separates higher- and lower-frequency movements in EPU, whereas SV-TVP-FAVAR estimates dynamic responses in a data-rich macroeconomic setting and allows those responses to differ across historical dates. Together, they address whether the EPU-medical inflation relationship differs across timescales and how the response of medical inflation changes across shock dates and quarterly horizons.

### 2.5 EPU-health studies and research gap

The empirical studies closest to the present paper connect policy uncertainty with health-sector outcomes. Cheng and Witvorapong (36) examine health-care policy uncertainty, real health expenditure, and health-care inflation in the United States using a Bayesian structural VAR. Their negative and persistent inflation responses show that the sign of the relationship can depend on the uncertainty measure and health-system context. For China, Bai et al. (37) use a spatial econometric framework and find that EPU is associated with healthcare expenditure and regional spillovers. Song et al. (38) examine monetary-policy effectiveness under post-COVID EPU and help characterize the wider policy-transmission environment. Sui et al. (39) apply an SV-TVP-FAVAR model to healthcare fiscal expenditure, making their method close to the present approach. These studies establish that uncertainty is relevant to health-sector resource flows, but their outcomes do not identify the price margin on which the present paper focuses. Healthcare expenditure combines prices, utilization, insurance coverage, demographics, and private payment, while fiscal expenditure primarily records public budget allocation and service provision. Medical inflation is therefore neither a substitute for those outcomes nor a relabeling of them; it isolates a different part of the health-system response.

Taken together, the literature leaves a specific problem unresolved. Public-health research explains why prices, insurance design, provider payment, procurement, and pharmaceutical policy matter for financial protection and access, while EPU research explains how policy uncertainty can alter macroeconomic behavior. The two bodies of work have rarely been joined to determine whether broad EPU enters China’s medical-price system. This separation matters for public health because the consequences of a price increase depend on where it occurs, how long it lasts, and whether insurance and public budgets can absorb it without shifting the burden to patients or constraining access. In particular, existing China-focused studies do not establish whether the relationship differs between higher- and lower-frequency components of EPU, whether it changes with the date of the shock and response horizon, or whether it is concentrated in medicines, services, or upstream pharmaceutical production. The present paper addresses this gap by treating medical inflation as a distinct health-system price outcome, separating EPU into higher- and lower-frequency components through wavelet filtering, and estimating time-varying responses to a one-standard-deviation EPU shock with an SV-TVP-FAVAR model and a large macroeconomic information set.

## 3 Materials and methods

### 3.1 Data sources and variables

The empirical analysis is based on quarterly Chinese data from 2004Q1 to 2026Q1. The uncertainty measure is the China EPU index based on the South China Morning Post (SCMP), obtained from the Economic Policy Uncertainty project and constructed within the newspaper-based framework of Baker et al. (1). The original monthly series is aggregated to quarters by taking the mean of the three monthly observations in each quarter. In the model, this series captures policy-related uncertainty as it appears in news coverage. Health-price variables and the macroeconomic information set come from Wind, which compiles official Chinese statistical releases, including series originally published by the National Bureau of Statistics of China. The included 2026Q1 observations may be revised in subsequent releases, so endpoint sensitivity is assessed by re-estimating the model on a sample ending in 2025Q4. Before time-series estimation, each variable is rendered stationary through logarithms or differencing according to the properties of that series. The main outcome is medical inflation, measured by the year-on-year growth rate of China’s CPI for health care.

The outcome choice is central to the design. Health expenditure growth combines prices, utilization, insurance coverage, demographic change, technology, and public spending. Medical inflation is narrower but more directly tied to the price pressure faced by households and payers. The baseline outcome is the year-on-year growth rate of China’s health-care CPI, which captures aggregate consumer-facing medical price pressure. The empirical analysis also examines two medicine-price component outcomes: the year-on-year growth rate of traditional Chinese medicine prices and the year-on-year growth rate of western medicine prices. These component outcomes allow the paper to assess whether medicine-price components display stronger EPU-related responses than the aggregate health-care CPI measure. The outcome-level robustness check and complementary production-side analysis then use two further health-sector price measures. The first is the year-on-year growth rate of medical service CPI, which isolates consumer-side medical service prices and excludes medicine-price components. The second is the year-on-year growth rate of pharmaceutical manufacturing PPI, which captures production-side and factory-gate price dynamics in pharmaceutical manufacturing.

The baseline sample contains 89 quarterly observations. Table 1 reports descriptive statistics for the core variables. The EPU index varies widely over the sample, consistent with several episodes of heightened policy uncertainty. The health-price series are not similar in either central tendency or volatility. Traditional Chinese medicine prices have the highest average growth rate and the widest range, whereas western medicine prices and pharmaceutical manufacturing PPI fluctuate around lower means. Medical service CPI remains positive throughout the aligned sample, whereas pharmaceutical manufacturing PPI exhibits both positive and negative movements. Because medical service CPI excludes medicine-price components, it provides an informative consumer-side check on whether the baseline pattern is driven only by medicine prices. These differences support the paper’s component-level design. Aggregate health-care CPI is the baseline measure of medical inflation, but it can mask heterogeneity between consumer-facing medicine-price and medical-service price responses. The separate pharmaceutical manufacturing PPI analysis extends the comparison to production-side pharmaceutical price dynamics. The Appendix reproduces the core-variable statistics with units and extends the descriptive overview to the full macroeconomic information set used for factor extraction.

**Table 1:** Descriptive statistics for core study variables.

Variable	*N*	Mean	SD	Excess kurtosis	Skewness	Min	Max
China news-based EPU index	89	277.211	245.062	−0.098	1.012	43.794	1011.689
Aggregate health-care CPI inflation	89	1.944	1.580	1.141	0.916	−1.000	6.913
Traditional Chinese medicine price inflation	89	3.856	3.994	0.377	0.395	−4.300	14.467
Western medicine price inflation	89	0.594	2.586	0.378	0.392	−5.567	6.300
Medical service CPI inflation	89	2.211	1.837	1.605	1.475	0.233	8.400
Pharmaceutical manufacturing PPI inflation	89	0.662	1.837	0.437	−0.088	−4.567	5.113

The sample covers quarterly observations from 2004Q1 to 2026Q1. EPU is the SCMP-based China index distributed through the Economic Policy Uncertainty project and constructed under the Baker et al. framework (1). All health-price variables are year-on-year growth rates. Supplementary material reports variable units, descriptive statistics, and the 2025Q4 endpoint-sensitivity analysis.

### 3.2 Macroeconomic information set

The SV-TVP-FAVAR model also relies on a large macroeconomic information set. The aim is to reduce the information loss that would arise in a small VAR. Medical inflation may move with real activity, investment, retail demand, external trade, aggregate prices, monetary and financial conditions, fiscal variables, health-related public spending, income, and demographics. A system containing only EPU and medical inflation would therefore risk omitting relevant macroeconomic conditions. After harmonization, the final information set contains 56 macroeconomic variables with sufficient quarterly observations. Three additional series broaden the coverage of input costs, health-sector demand, and financing conditions: West Texas Intermediate (WTI) crude-oil prices, retail sales of traditional Chinese and western medicines, and aggregate financing to the real economy (AFRE, flow). WTI prices proxy transport and petroleum-based medical input costs (40); medicine retail sales proxy health-sector demand; and AFRE captures financing conditions beyond bank lending. The full set covers output, real activity, aggregate prices, trade, money and credit, exchange rates, capital-market conditions, fiscal variables, health-related public spending, business-cycle indicators, household income, inequality, and demographics. The goal is to approximate the environment in which health-sector prices are formed. After the stationarity transformations, the variables are standardized so that units and measurement scales do not dominate the principal-component estimates. Three latent common factors are extracted from this standardized information set by principal component analysis, following Bernanke et al. (35). Appendix Table A2 reports descriptive statistics for the full information set.

### 3.3 Empirical strategy

#### 3.3.1 Rationale for a multi-scale and time-varying framework

The research question is not well served by either a static correlation or a constant-parameter VAR. A static correlation would collapse the EPU-medical inflation relation into one sample average. A constant-parameter VAR would impose stable dynamics and stable shock variances throughout the sample. These assumptions are restrictive for a sample that includes the global financial crisis, China’s policy adjustments, COVID-19, and post-pandemic health-system and pharmaceutical-market changes. The methods are chosen to match that setting. Wavelet filtering separates higher- and lower-frequency components of EPU and asks whether they generate different medical-inflation responses. The SV-TVP-FAVAR model then traces these responses across shock dates and response horizons while allowing dynamic transmission and shock volatility to evolve. The two tools are complementary: wavelet analysis describes time-frequency heterogeneity, while SV-TVP-FAVAR traces dynamic shock responses in a data-rich macroeconomic environment.

#### 3.3.2 Wavelet-based multi-scale analysis

The wavelet stage applies the three-level Haar maximal overlap discrete wavelet transform (MODWT) to EPU in MATLAB with periodic boundary extension, yielding three detail components and one terminal approximation component. For quarterly data, D1 corresponds approximately to fluctuations with periods of 2–4 quarters, D2 to 4–8 quarters, D3 to 8–16 quarters, and the terminal approximation component A3 to movements longer than 16 quarters. D1 captures the shortest-term disturbances, D2 short cyclical movements, D3 medium-term variation, and A3 longer-run movements. This partition makes the frequency content explicit before the time-varying VAR is estimated. Only EPU is filtered in the frequency-specific models; the medical-price outcome remains unfiltered.

Let $x_{t}$ denote the EPU series. The decomposition used in the frequency-specific models is given by Equation 1:


$$x_{t}=H_{t}+L_{t}$$ (1)

where the higher-frequency component is $H_{t}=D_{1,t}+D_{2,t}$ , corresponding approximately to movements of 2–8 quarters, and the lower-frequency component is $L_{t}=D_{3,t}+A_{3,t}$ , corresponding to movements longer than 8 quarters. Here, D1, D2, and D3 denote the three Haar detail components, and A3 denotes the terminal approximation component from the three-level decomposition. The two constructed series distinguish higher- from lower-frequency movements in EPU. In the frequency-specific models, $H_{t}$ and $L_{t}$ replace the aggregate EPU series as the uncertainty input to the otherwise unchanged SV-TVP-FAVAR system.

This decomposition of EPU allows the empirical analysis to ask whether medical inflation responds differently to shorter-cycle and more persistent components of policy uncertainty. The quarterly frequency bands generated by the three-level Haar decomposition are used consistently in the frequency-specific response analysis.

#### 3.3.3 SV-TVP-FAVAR model

To study the dynamic effect of EPU shocks on medical inflation, it is useful to begin with a basic VAR system linking EPU to medical inflation. In a small constant-parameter VAR, however, two limitations arise. First, the relationship between EPU and medical inflation is forced to remain constant over the full sample. This is restrictive in a setting that includes major crisis episodes, post-pandemic adjustments, and health-sector policy changes. Second, a small VAR can include only a limited number of variables, even though medical inflation is shaped by a wider macroeconomic environment involving real activity, monetary and financial conditions, producer prices, fiscal pressure, and health-sector demand. A small system may therefore omit information that matters for the response of medical prices.

The empirical model therefore extends the basic VAR in three steps, following the logic of factor-augmented and time-varying VAR applications in macroeconomic policy analysis (33–35, 41, 42). First, we extract latent common factors from a large macroeconomic information set using principal component analysis after transforming the variables to stationarity and standardizing them. This factor-augmentation step retains broad information while avoiding the dimensionality problem that would arise if all macroeconomic indicators entered the VAR directly. Second, we allow the VAR coefficients to vary over time, so the effect of EPU shocks on medical inflation can differ across historical periods. Third, we allow the covariance matrix of innovations to evolve through stochastic volatility, because crisis periods may involve larger and more clustered shocks than normal periods. The resulting model is a stochastic-volatility time-varying parameter factor-augmented VAR, or SV-TVP-FAVAR.

Let $X_{t}$ denote an N×1 vector of observed macroeconomic and financial variables, and let $F_{t}$ be a K×1 vector of latent common factors extracted from $X_{t}$ , where K≪N . In this study, K=3 . Let $p_{t}$ denote the health-price outcome, with aggregate medical inflation as the baseline outcome, and let $u_{t}$ denote the EPU index. The M×1 state vector entering the VAR system, with M=K+2 , is defined by Equation 2:


$$Y_{t}={\left[u_{t},{F_{t}}^{'},p_{t}\right]}^{'}$$ (2)

The observation equation for the information set is given by Equation 3:


$$X_{t}=\Lambda_{F}F_{t}+\lambda_{p}p_{t}+\lambda_{u}u_{t}+e_{t},$$ (3)

where $\Lambda_{F}$ is the N×K factor-loading matrix, $\lambda_{p}$ and $\lambda_{u}$ are N×1 loading vectors for medical inflation and EPU, and $e_{t}$ is an N×1 vector of idiosyncratic errors. This observation equation links the large information set to a smaller state vector containing the extracted common factors, medical inflation, and EPU. The time-varying VAR system is specified by Equations 4,5:


$$Y_{t}=c_{t}+\sum_{j=1}^{q}B_{j,t}Y_{t-j}+\varepsilon_{t}$$ (4)

$$\varepsilon_{t}\sim N(0,\Omega_{t}).$$ (5)

The lag length is selected by the Akaike information criterion, which yields one lag in the empirical implementation. The first two, three, and four principal components explain 78.6, 87.9, and 89.3% of the information-set variance, respectively. The baseline uses three factors because the fourth adds only 1.4 percentage points of explained variance while increasing the time-varying system’s dimension. The model computes impulse responses to a one-standard-deviation innovation in EPU over horizons of 1 to 15 quarters. Following the time-varying structural VAR framework of Primiceri (33), and taking into account the corrected stochastic-volatility estimation procedure discussed by Del Negro and Primiceri (34), both the VAR coefficients and the variance–covariance matrix of innovations are allowed to evolve over time. The system is estimated by MCMC-based Bayesian methods. Gamma priors with shape 40 and rate 0.02 are assigned to the innovation-variance precisions of the time-varying VAR coefficients, contemporaneous coefficients, and log-volatility states; initial states have mean zero and covariance equal to ten times the identity matrix. Estimation uses 10,000 iterations, 1,000 burn-in iterations, no thinning, and 9,000 retained draws. For five monitored state-innovation parameters, absolute Geweke CD statistics range from 0.025 to 0.891 and inefficiency factors from 5.283 to 73.140. For every shock date and response horizon, the posterior distribution is summarized by its mean and 16th and 84th percentiles, which define a central 68% credible interval. Figures 1–7 display the posterior-mean surfaces, and Figure 8 shows an eight-quarter horizon slice with posterior bands. Supplementary Table S2 reports P16, the posterior mean, and P84 at the full-sample posterior-mean maximum for each response specification, while Supplementary Data S2 provides the corresponding posterior summaries for every shock-date/horizon cell.

**Figure 1:**
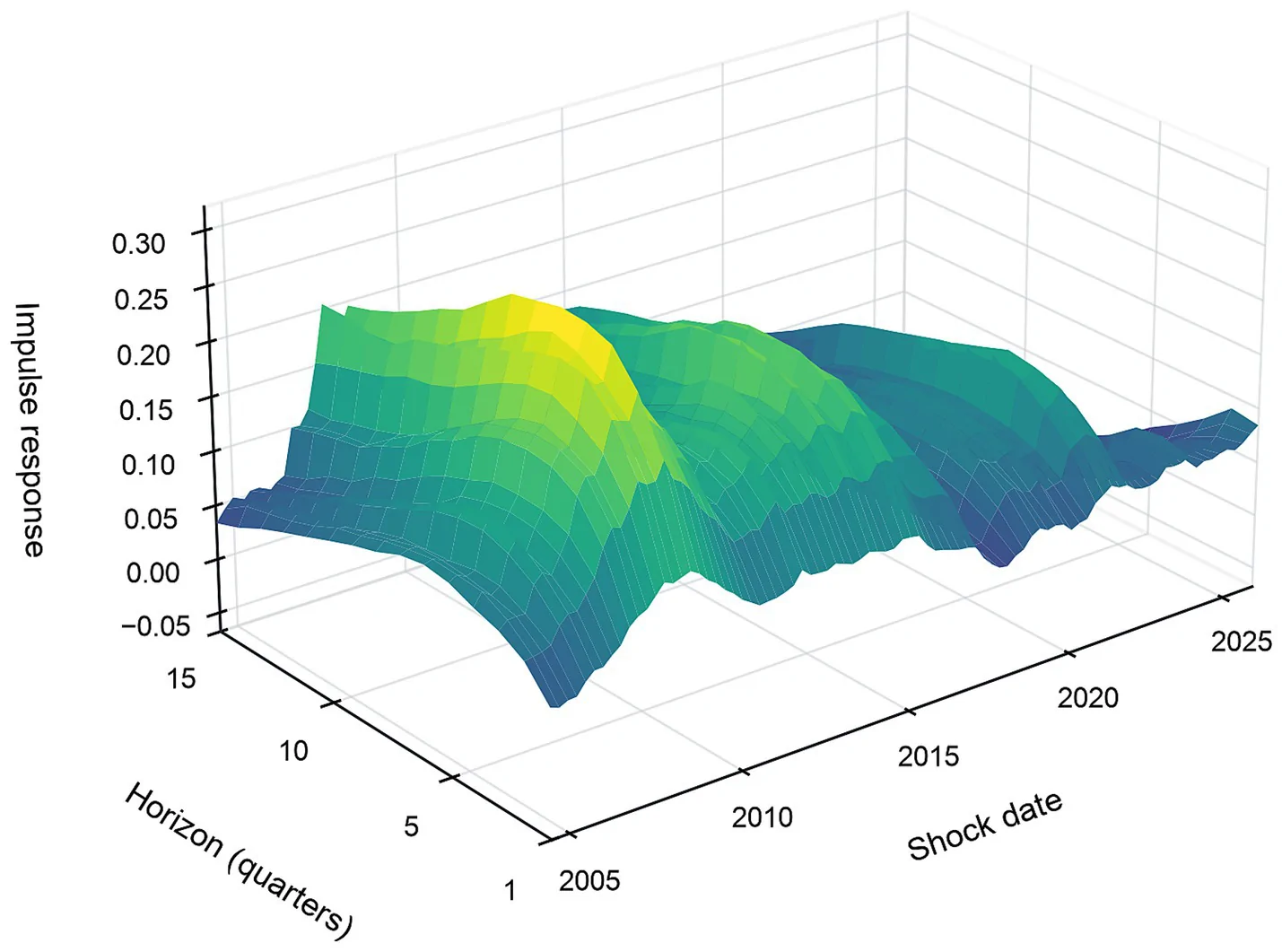
Posterior-mean response of medical inflation to a one-standard-deviation shock to the higher-frequency EPU component. The filtered EPU component combines Haar D1 and D2 (approximately 2–8 quarters). Axes report shock date, response horizon in quarters, and impulse response. P16 and P84 at the reported posterior-mean maximum are provided in Supplementary Table S2; complete cell-level posterior summaries are available in Supplementary Data S2.

**Figure 2:**
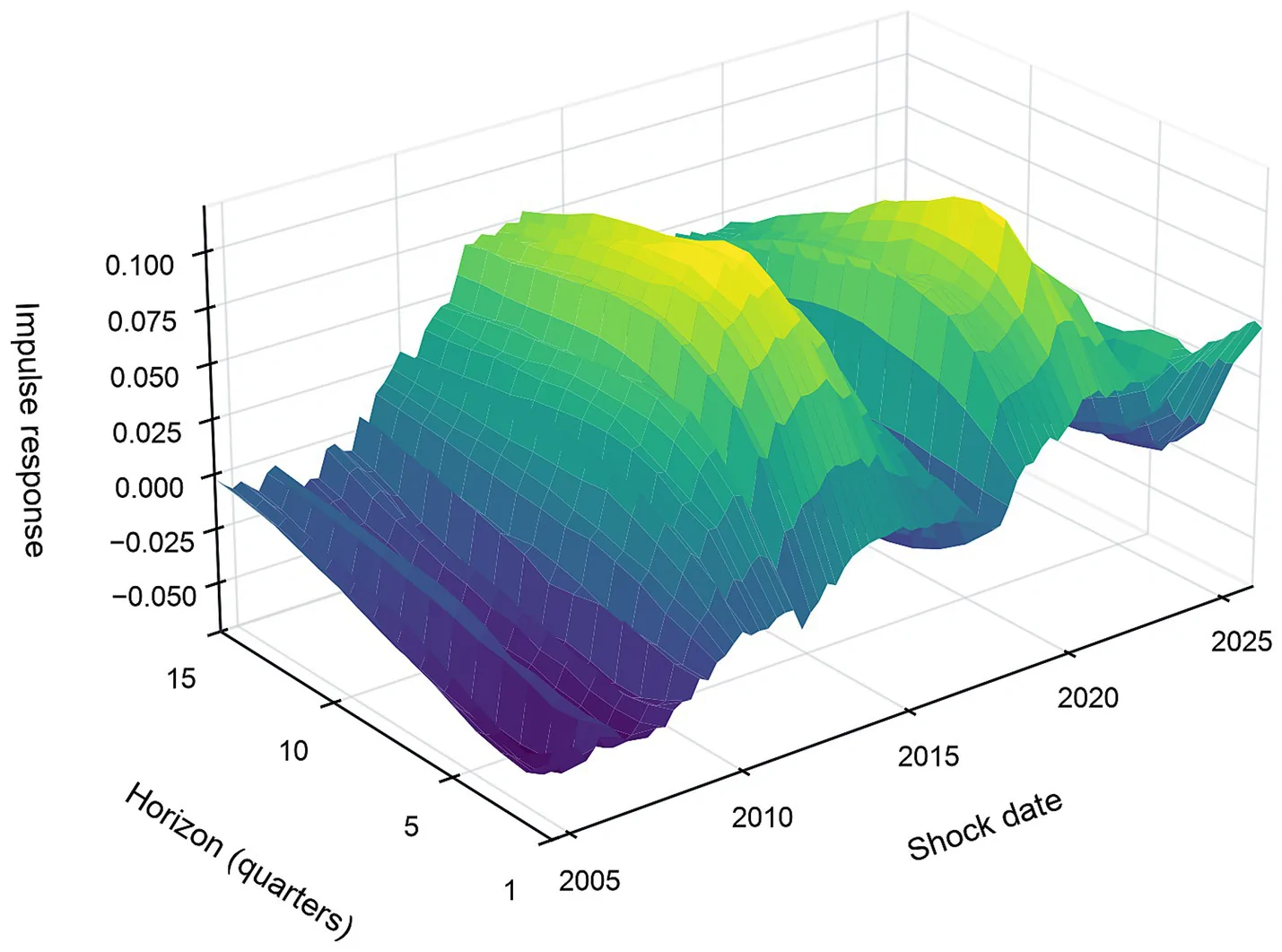
Posterior-mean response of medical inflation to a one-standard-deviation shock to the lower-frequency EPU component. The filtered EPU component combines Haar D3 and the terminal approximation component A3 (more than 8 quarters). Axes report shock date, response horizon in quarters, and impulse response. P16 and P84 at the reported posterior-mean maximum are provided in Supplementary Table S2; complete cell-level posterior summaries are available in Supplementary Data S2.

**Figure 3:**
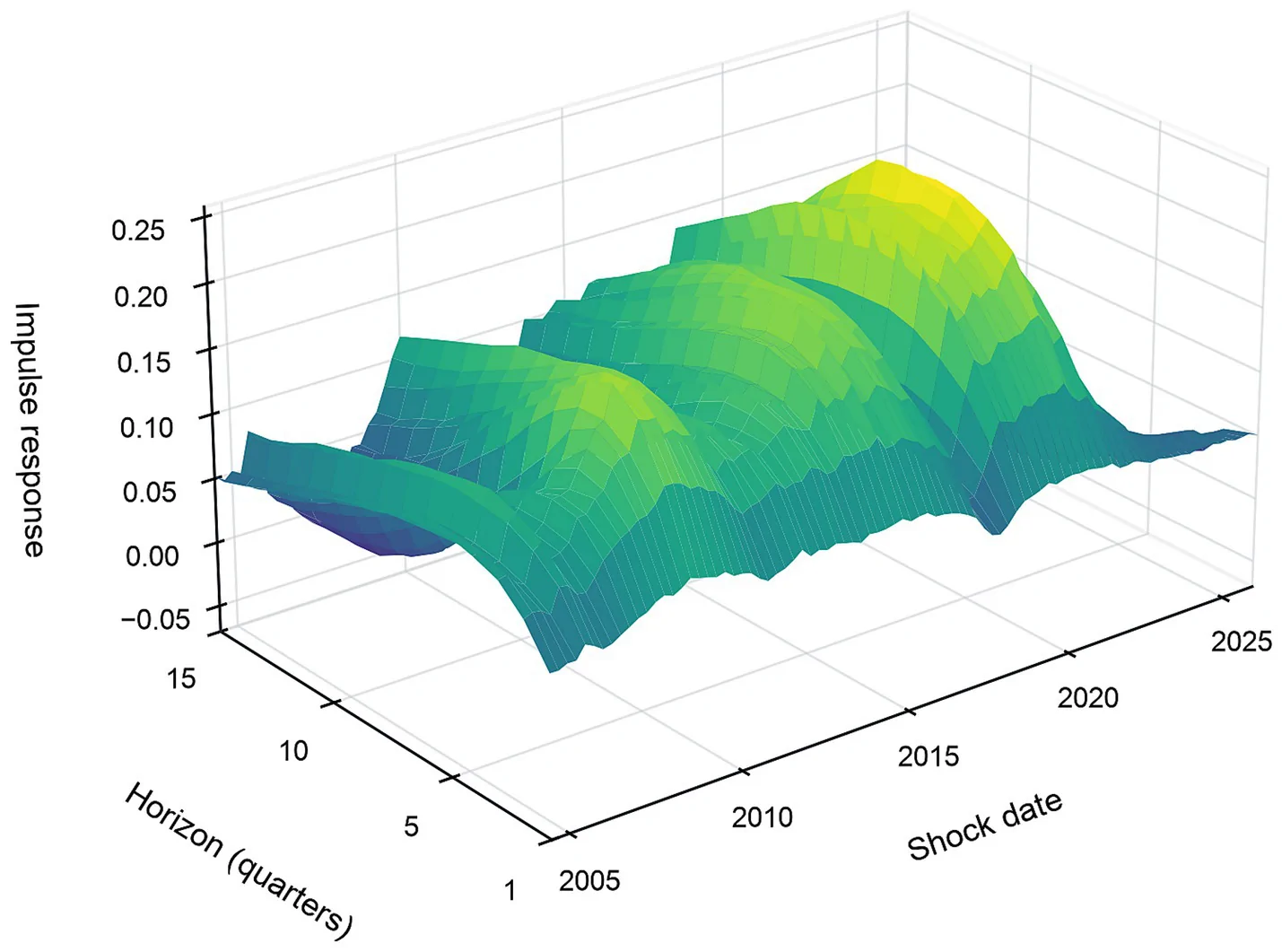
Posterior-mean response of aggregate health-care CPI to a one-standard-deviation EPU shock. Axes report shock date, response horizon in quarters, and impulse response. P16 and P84 at the reported posterior-mean maximum are provided in Supplementary Table S2; complete cell-level posterior summaries are available in Supplementary Data S2.

**Figure 4:**
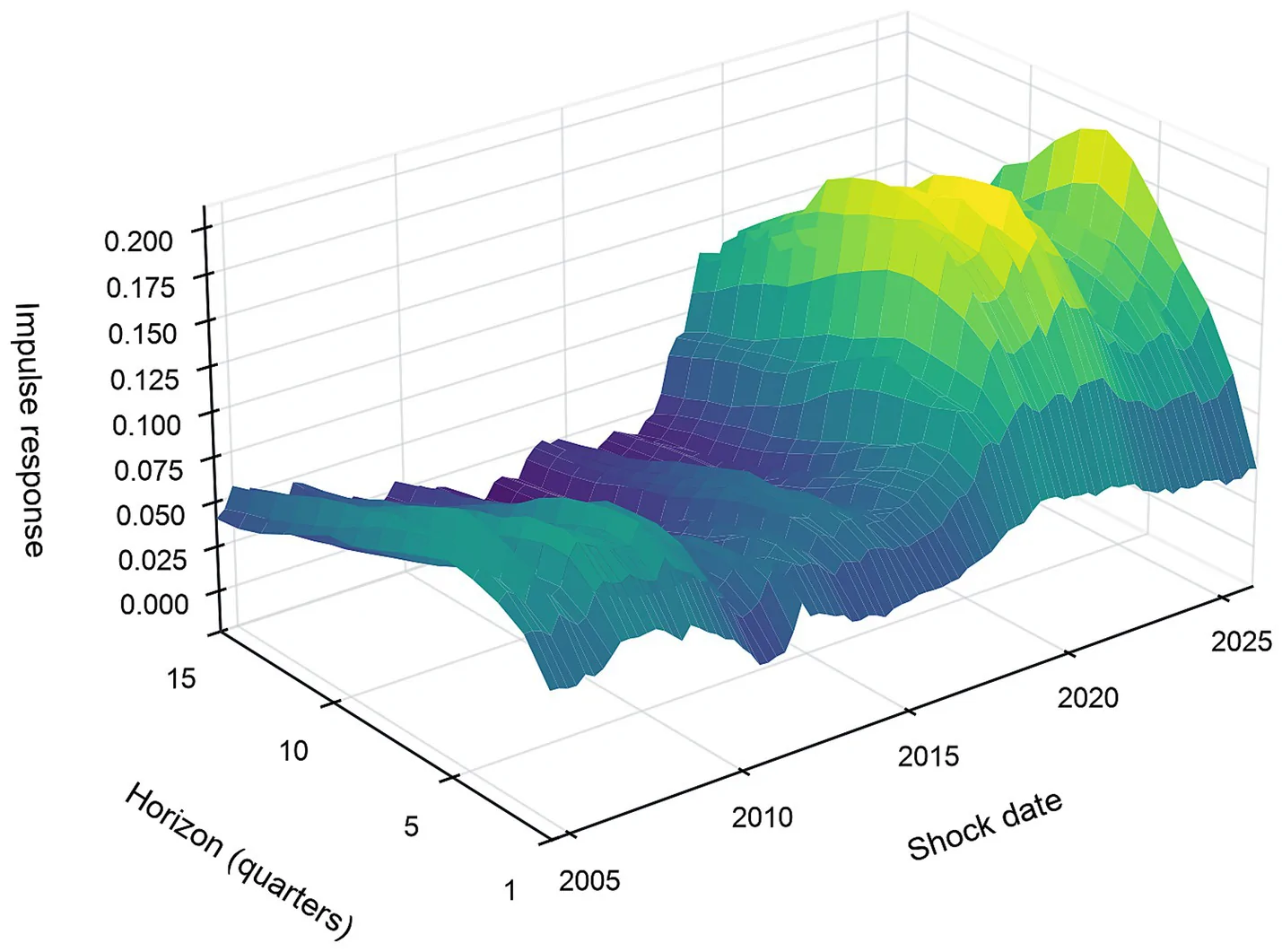
Posterior-mean response of traditional Chinese medicine prices to a one-standard-deviation EPU shock. Axes report shock date, response horizon in quarters, and impulse response. P16 and P84 at the reported posterior-mean maximum are provided in Supplementary Table S2; complete cell-level posterior summaries are available in Supplementary Data S2.

**Figure 5:**
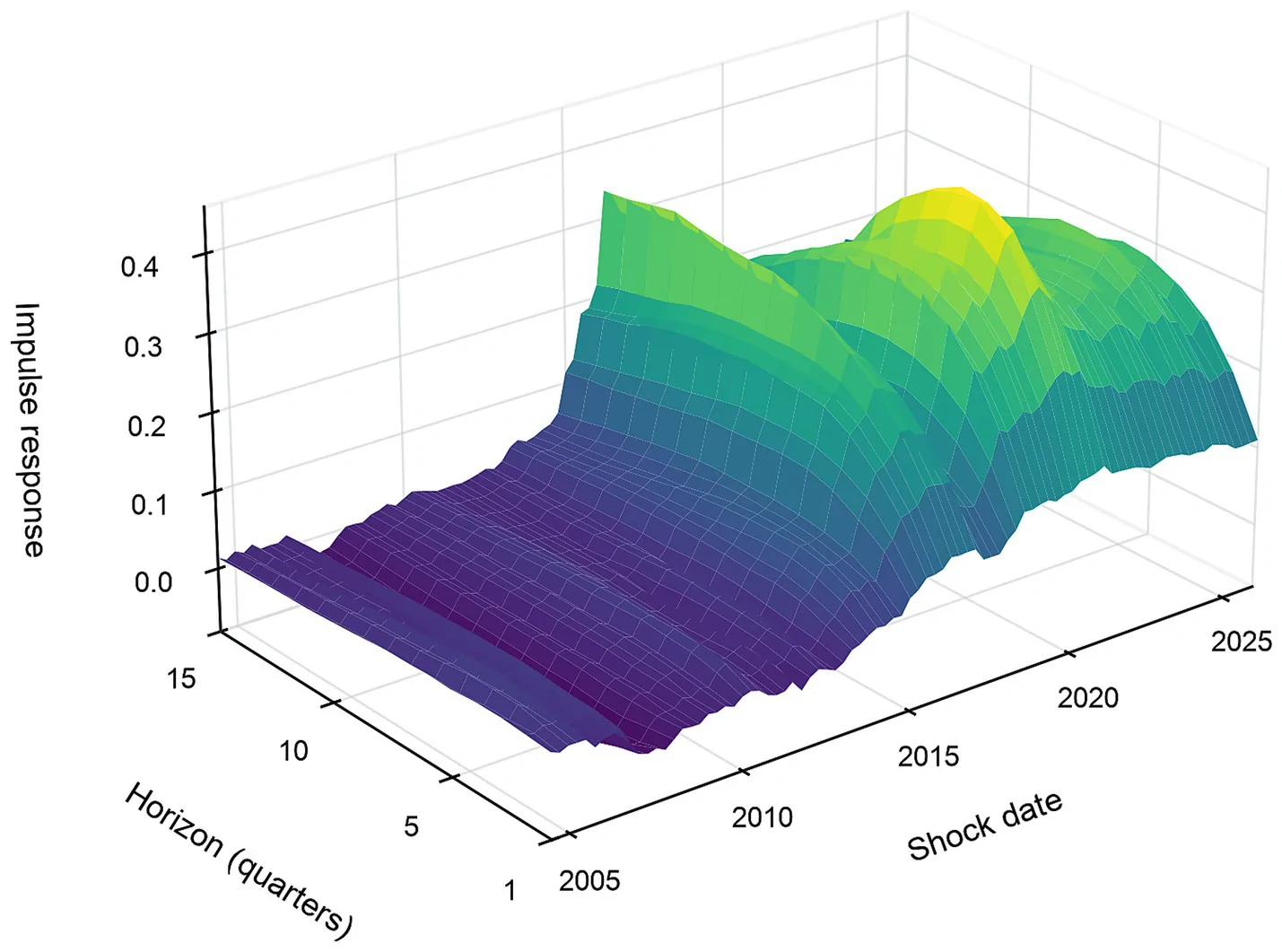
Posterior-mean response of western medicine prices to a one-standard-deviation EPU shock. Axes report shock date, response horizon in quarters, and impulse response. P16 and P84 at the reported posterior-mean maximum are provided in Supplementary Table S2; complete cell-level posterior summaries are available in Supplementary Data S2.

**Figure 6:**
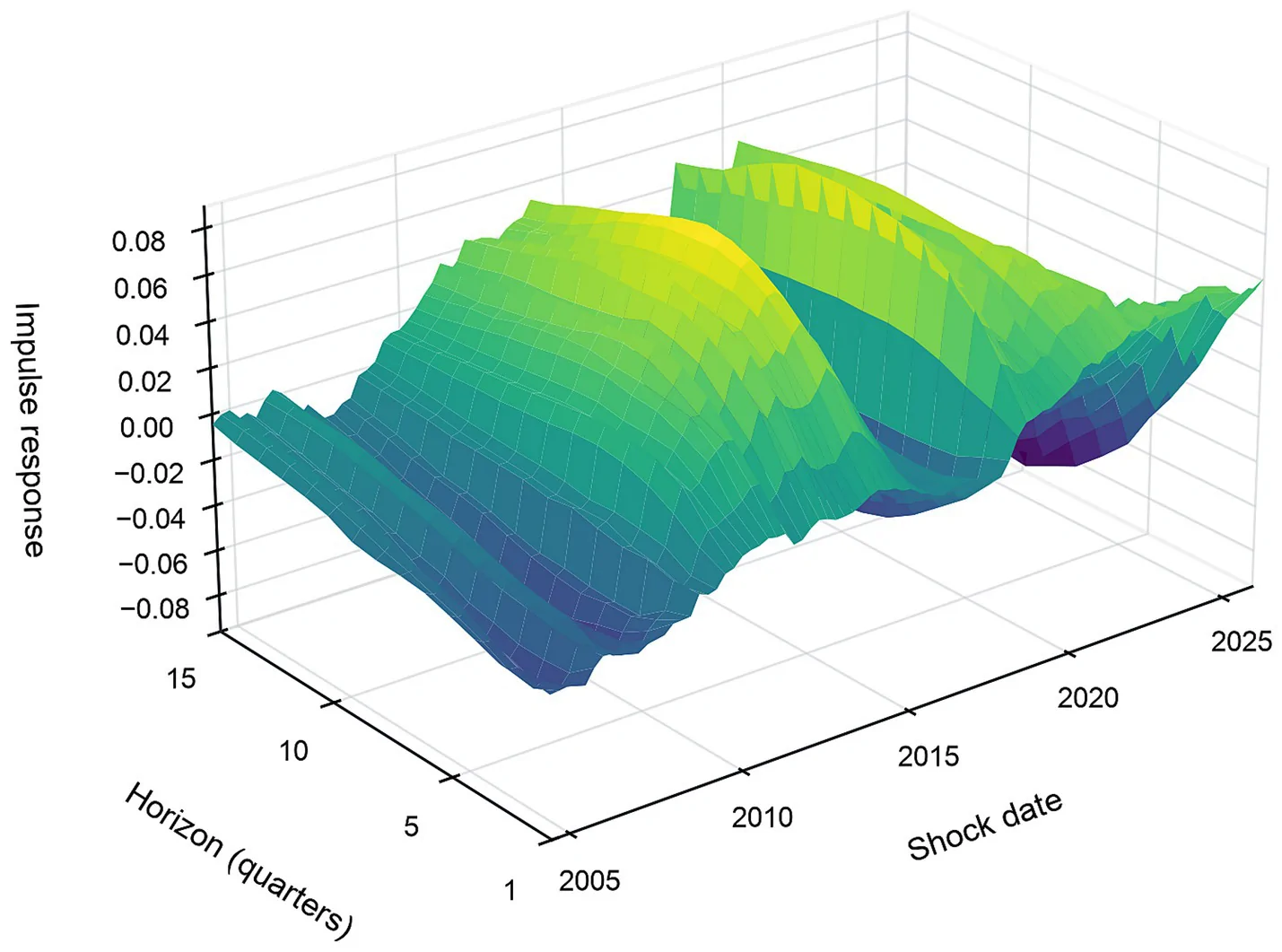
Posterior-mean response of medical service CPI to a one-standard-deviation EPU shock. Axes report shock date, response horizon in quarters, and impulse response. P16 and P84 at the reported posterior-mean maximum are provided in Supplementary Table S2; complete cell-level posterior summaries are available in Supplementary Data S2.

**Figure 7:**
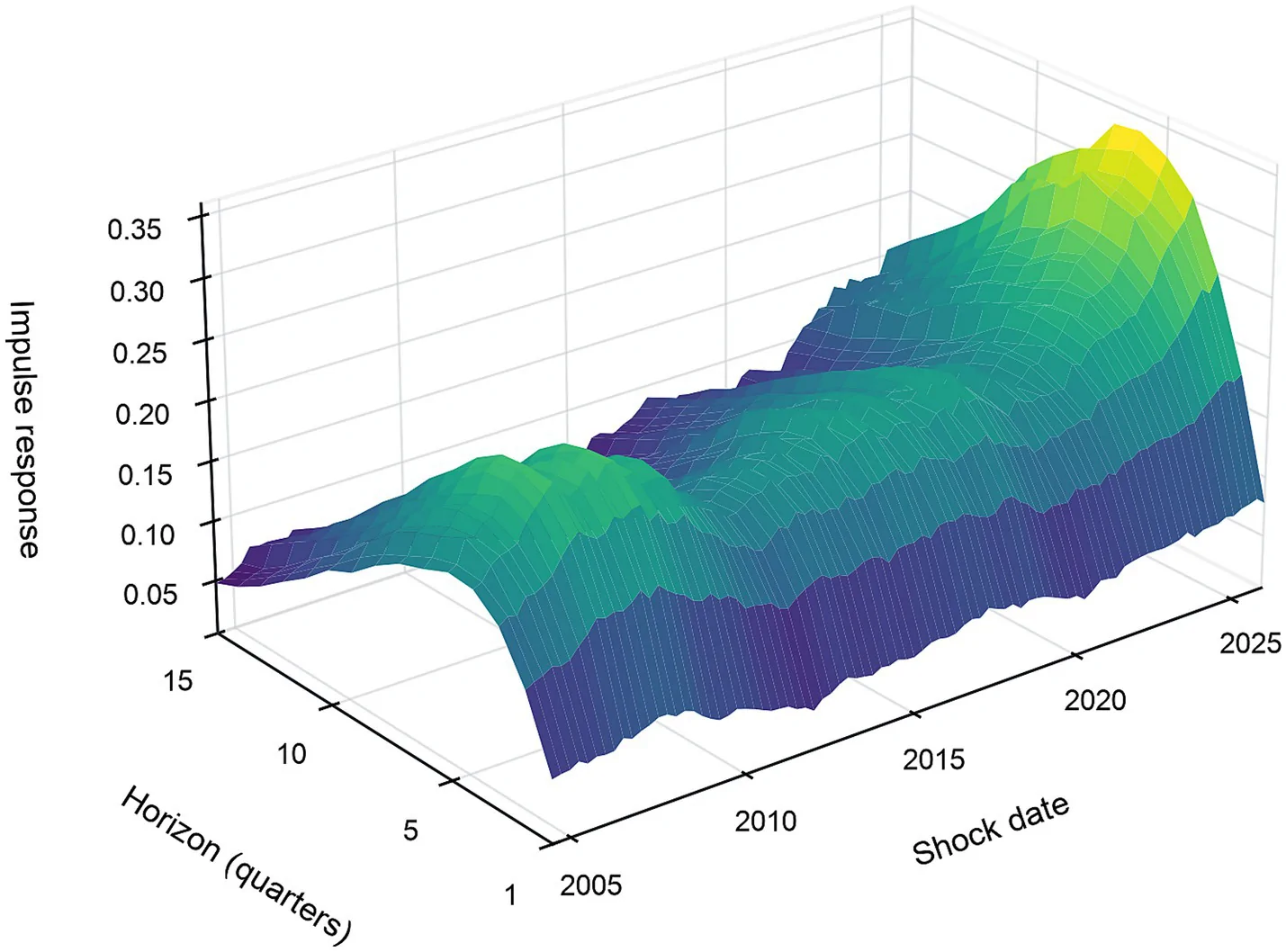
Posterior-mean response of pharmaceutical manufacturing PPI to a one-standard-deviation EPU shock. Axes report shock date, response horizon in quarters, and impulse response. P16 and P84 at the reported posterior-mean maximum are provided in Supplementary Table S2; complete cell-level posterior summaries are available in Supplementary Data S2.

**Figure 8:**
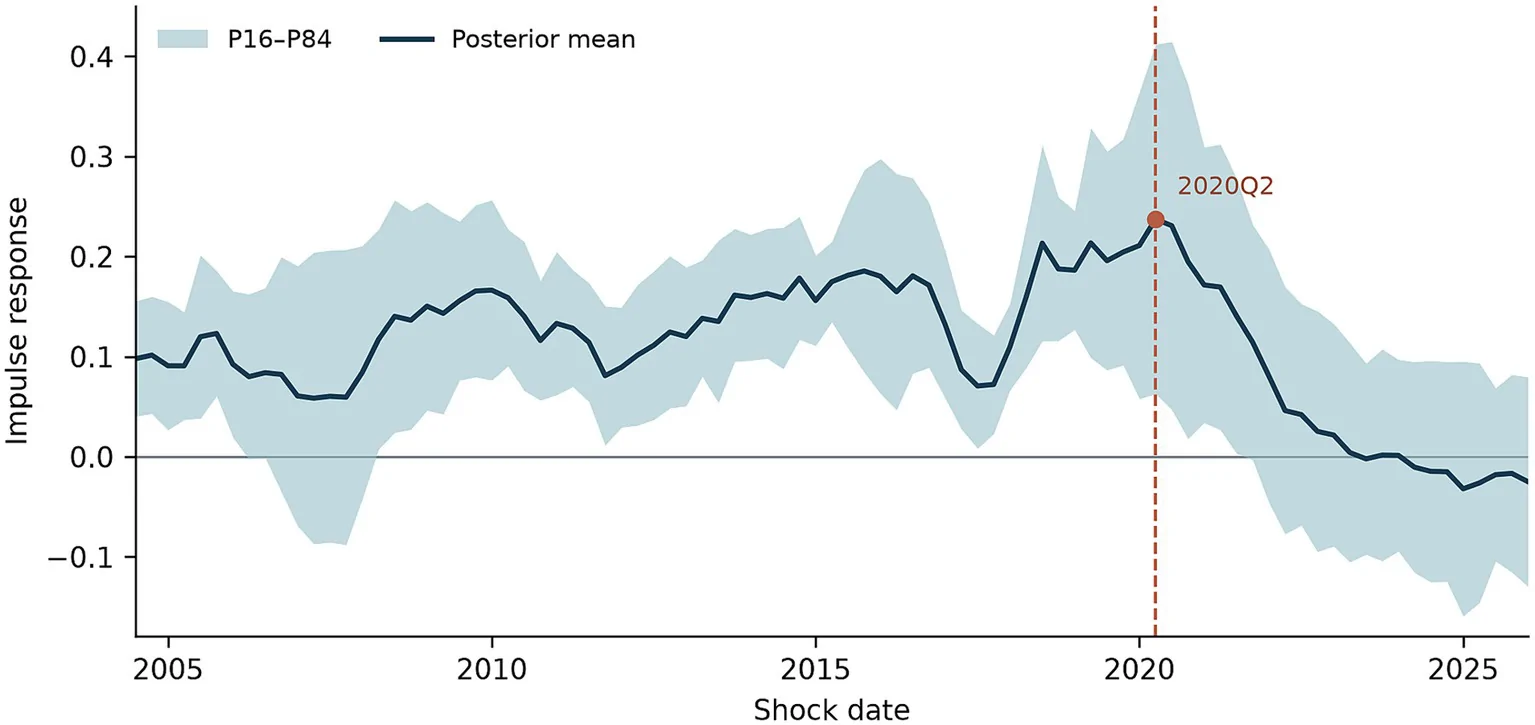
Aggregate health-care CPI response at an eight-quarter horizon across EPU shock dates. The line reports the posterior mean and the shaded region the 16th–84th percentile posterior interval. The vertical guide marks 2020Q2. The horizontal axis reports the shock date and the vertical axis the impulse response to a one-standard-deviation EPU shock.

The time-varying covariance matrix can be decomposed as shown in Equation 6:


$$A_{t}\Omega_{t}A_{t}^{'}=\Sigma_{t}\Sigma_{t}^{'},$$ (6)

Equivalently, Equation 7 gives:


$$\Omega_{t}=A_{t}^{-1}\Sigma_{t}\Sigma_{t}^{'}{\left(A_{t}^{-1}\right)}^{'},$$ (7)

where $A_{t}$ is a lower triangular M×M matrix with unit diagonal elements, and $\Sigma_{t}=\mathrm{diag}(\sigma_{1t},\ldots ,\sigma_{\mathrm{Mt}})$ is a diagonal matrix containing time-varying standard deviations. Let $\beta_{t}=\mathrm{vec}(c_{t},B_{1,t},\ldots ,B_{q,t})$ collect the time-varying VAR coefficients, let $\alpha_{t}$ collect the free lower-triangular elements of $A_{t}$ , and let $h_{t}={\left(\log\sigma_{1t},\ldots ,\log\sigma_{\mathrm{Mt}}\right)}^{'}$ collect the log-volatilities. The model allows these state variables to follow the random-walk processes in Equations 8−10:


$$\beta_{t}=\beta_{t-1}+\upsilon_{t},$$ (8)

$$\alpha_{t}=\alpha_{t-1}+\varsigma_{t},$$ (9)

$$h_{t}=h_{t-1}+\eta_{t}.$$ (10)

Structural innovations are identified recursively through the lower-triangular contemporaneous matrix $A_{t}$ . Equation 2 orders the state vector as $Y_{t}={\left[u_{t},{F_{t}}^{'},p_{t}\right]}^{'},$ where $u_{t}$ denotes EPU, $F_{t}$ contains the three common factors, and $p_{t}$ denotes the health-price outcome. Equivalently, the five variables follow the recursive ordering (EPU, F1, F2, F3, MI). Under this Cholesky identification, an EPU innovation can affect the common factors and medical inflation contemporaneously within the quarter, whereas innovations ordered later affect EPU only with a lag. Placing the factors before medical inflation allows broad macro-financial innovations to affect the health-price outcome contemporaneously. The same ordering is used in the baseline and component-specific models, and lagged interactions among all variables remain unrestricted.

This structure allows the transmission of EPU shocks to medical inflation to vary across historical dates and quarterly response horizons. In Figures 1–7, the horizontal axes identify the shock date and the response horizon from 1 to 15 quarters, while the vertical axis reports the posterior-mean impulse response to a one-standard-deviation EPU innovation. The corresponding P16-P84 posterior intervals are reported in Supplementary Table S2 and Supplementary Data S2.

For a given historical date t and response horizon h , the time-varying impulse response of medical inflation to an EPU shock with can be summarized by Equation 11:


$${\mathrm{IRF}}_{p,t}(h)=E_{t}[p_{t+h}|\varepsilon_{u,t}=\sigma_{u,t}]-E_{t}[p_{t+h}|\varepsilon_{u,t}=0]$$ (11)

where h=1,…,15 , $\varepsilon_{u,t}$ denotes the structural EPU innovation and $\sigma_{u,t}$ denotes its time-varying standard deviation. This notation matches the empirical figures, which report responses to a one-standard-deviation EPU shock over quarterly horizons.

## 4 Results

### 4.1 Extracted common factors

Figure 9 reports the three common factors extracted from the macroeconomic information set. Their movements line up with the main macroeconomic and policy episodes in the sample. The first factor rises over 2018–2020, then falls back and edges up again near the end. The second rises markedly during the global financial crisis, with additional movements around 2015 and 2020. The third is elevated in 2008 and becomes uneven again around 2020 and 2024–2025. Figure 9 also shows why factor augmentation is useful. Instead of relying on a narrow set of observed controls, the model uses a small number of common factors to summarize broader macro-financial conditions. The broader macroeconomic setting matters because medical inflation may move with general activity, fiscal pressure, monetary conditions, production costs, and health-sector demand. A narrow system containing only EPU and medical inflation would risk treating those background movements as part of the uncertainty response. By embedding the EPU-medical inflation relationship in a factor-augmented framework, the model uses the common factors to absorb broad macro-financial conditions and reduces the risk that omitted macroeconomic variation is mistakenly attributed to an EPU shock.

**Figure 9:**
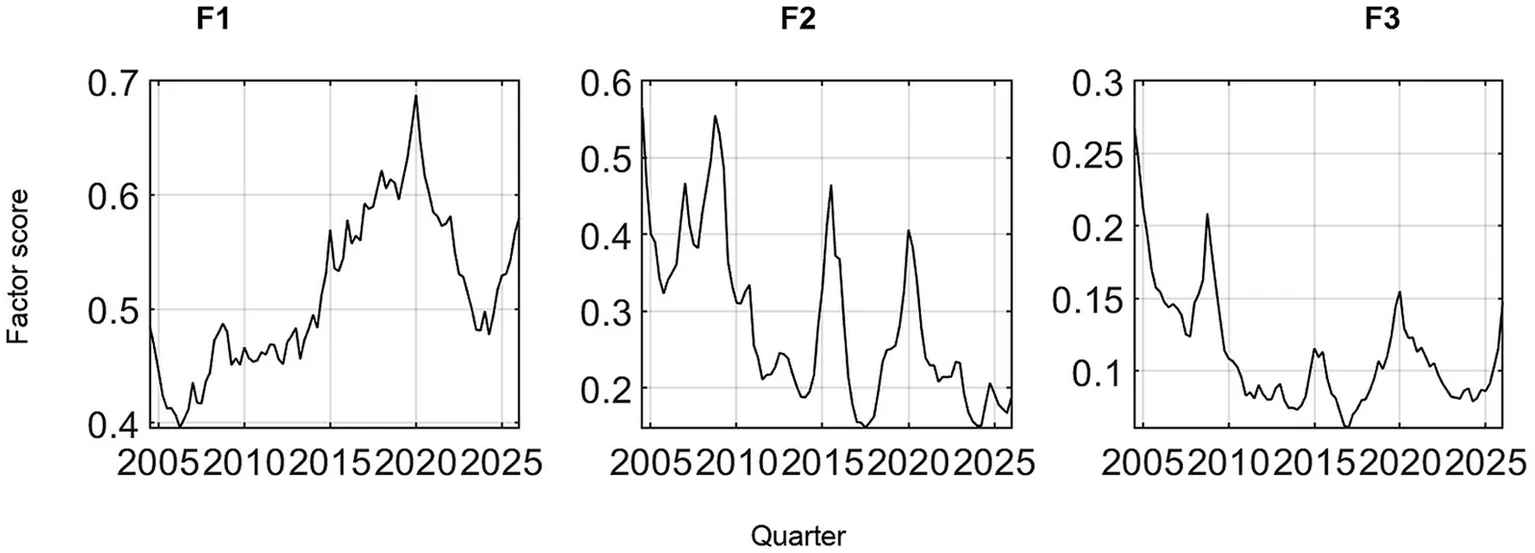
Extracted common factors. The horizontal axis reports quarter and the vertical axis the factor score. Panels are labeled F1, F2, and F3.

### 4.2 Responses to higher- and lower-frequency EPU components

Figure 1 reports the response to the higher-frequency EPU component, corresponding approximately to movements of 2–8 quarters. Its posterior-mean maximum is 0.297 for shocks dated 2008Q3 at a six-quarter horizon; the corresponding 16th and 84th posterior percentiles are 0.122 and 0.472. Figure 2 reports the response to the lower-frequency EPU component, corresponding to movements longer than eight quarters. Its posterior-mean maximum is 0.108 for shocks dated 2013Q2 at a seven-quarter horizon, with a central 68% credible interval of [0.035, 0.181]. Both frequency-specific baseline models display positive responses, with a larger full-sample maximum for the higher-frequency EPU component. In the sample ending in 2025Q4, their maxima are 0.214 and 0.208, respectively.

The full-sample posterior-mean maximum is larger for the higher-frequency EPU component, although the endpoint-sensitivity analysis substantially narrows the difference. Higher-frequency uncertainty may reflect shorter-cycle policy news, financing disturbances, and revisions to near-term expectations. Lower-frequency uncertainty may reflect more persistent changes in expectations surrounding procurement, reimbursement, and administered pricing. The maxima occur at different shock dates and response horizons: the higher-frequency maximum is associated with the global financial crisis, whereas the lower-frequency maximum occurs later and one quarter further into the response window. At each reported maximum, the central 68% credible interval lies above zero. Together, the results indicate that medical-price responses are present at both EPU frequency scales. The following sections examine how these responses differ across aggregate health-care CPI, medicine prices, medical services, and pharmaceutical manufacturing.

### 4.3 Baseline dynamic response of aggregate medical inflation

Figure 3 turns from frequency components to aggregate health-care CPI. The posterior-mean response is positive but varies across shock dates and horizons. It reaches 0.237 for a shock dated 2020Q2 at an eight-quarter horizon, with a central 68% credible interval of [0.063, 0.412]. Figure 8 plots the eight-quarter posterior mean and P16–P84 band across shock dates. The posterior mean is elevated around 2020Q2, but its band overlaps those at earlier comparison dates, including 2010Q2 and 2014Q2. The timing coincides with the first phase of COVID-19, when health-service use, supply conditions, procurement, and policy implementation were undergoing unusually large adjustments. The broader surface also shows why the response should not be summarized by a single full-sample coefficient: shocks dated in different periods produce different horizon profiles. As the broadest consumer-facing health-price measure, health-care CPI serves as the benchmark for the component results. It combines medicine prices, medical service prices, and other health-care price movements in one index, so a moderate aggregate response can coexist with sharper adjustment in one part of the basket and weaker movement elsewhere. This distinction is important for health economics because the same aggregate inflation rate can arise from different combinations of pharmaceutical and service-price pressure, with different implications for households, providers, and public payers. The component analysis examines heterogeneity across medical-price categories and identifies where the estimated price response is most pronounced within the health-price basket.

### 4.4 Heterogeneity across medicine-price components

Figure 4 shows a positive but comparatively moderate response for traditional Chinese medicine prices. The posterior-mean maximum is 0.197 for a shock dated 2021Q2 at a five-quarter horizon, with a central 68% credible interval of [0.126, 0.268]. This maximum is below the aggregate health-care CPI maximum of 0.237, indicating a smaller consumer-side response than the aggregate benchmark. Traditional Chinese medicine prices are exposed to raw-material supply, seasonal availability, procurement conditions, distribution costs, and policy expectations that need not move with the rest of the health-price basket. The component-level result therefore identifies a distinct positive response while separating price adjustment from changes in utilization and public spending.

In the baseline full-sample specification, Figure 5 displays the largest consumer-side posterior-mean maximum. Western medicine prices reach a posterior-mean maximum of 0.424 for a shock dated 2020Q3 at a six-quarter horizon, with a central 68% credible interval of [0.231, 0.618]. The maximum is about 79% larger than the aggregate health-care CPI maximum and more than twice the traditional Chinese medicine maximum. A broader elevated region after 2020 is also visible on the posterior-mean surface. This pattern is consistent with the relevance of manufacturing costs, import conditions, procurement rules, regulation, distribution networks, and expectations concerning future health policy. Figures 4, 5 also reveal substantial heterogeneity within medicine prices. The two components face different input markets and institutional arrangements, and their estimated responses differ substantially in both magnitude and timing. In this specification, western medicine prices show the largest consumer-side posterior-mean response, while the traditional Chinese medicine response remains positive but more moderate.

The component results also place the findings in the wider uncertainty-inflation literature. Under a predominantly demand-side mechanism, greater uncertainty would be expected to weaken spending and reduce inflationary pressure. The positive medicine-price responses are consistent with a role for supply conditions and policy expectations in the health sector, where pharmaceutical prices are shaped by production costs, procurement arrangements, regulation, distribution networks, and expectations about future payment rules. This distinction is important from a health-economics perspective. The closest China-focused EPU-health studies examine health-care spending or fiscal expenditure, whereas the present analysis follows price adjustment within the medical-price system itself. The component results reveal substantial heterogeneity within that system: western medicine prices display the largest consumer-side baseline maximum, traditional Chinese medicine prices respond more moderately, and the aggregate health-care CPI combines these movements with medical services and other health-care prices. The evidence therefore shows not only that EPU is associated with medical inflation, but also how estimated price adjustment differs within the health-price basket.

### 4.5 Robustness checks and additional analyses

Figure 6 replaces aggregate health-care CPI with medical service CPI. The posterior-mean maximum is 0.078 for a shock dated 2014Q1 at an eight-quarter horizon, with a central 68% credible interval of [0.046, 0.109]. Because this outcome excludes medicine prices, it provides a consumer-side comparison outside the pharmaceutical components. Its smaller magnitude highlights heterogeneity within the health-price basket and is consistent with medical service prices being more directly shaped by provider payment schedules, administrative rules, and reimbursement arrangements. Figure 7 then turns to pharmaceutical manufacturing PPI, a factory-gate rather than consumer-facing price measure. Its posterior-mean maximum is 0.338 for a shock dated 2026Q1 at a six-quarter horizon, with a central 68% credible interval of [0.115, 0.561]. The 2026Q1 maximum is at the final shock date and is not reproduced when the sample ends in 2025Q4; it is likely an endpoint effect rather than a stable production-side peak. The production-side surface reflects a different stage of price formation, where input costs, inventories, import conditions, and supply-chain adjustment can affect factory-gate prices independently of contemporaneous movements in consumer-facing medical prices.

To assess sensitivity to the most recent observation, all seven response models were re-estimated on a sample ending in 2025Q4 while retaining the same model structure and 1-15-quarter response window. The aggregate health-care CPI peak remains associated with a 2020Q2 shock, with the posterior-mean maximum changing from 0.237 at an eight-quarter horizon in the full sample to 0.376 at an eleven-quarter horizon in the truncated sample. The maximum for the higher-frequency EPU component changes from 0.297 to 0.214 and remains associated with 2008Q3, whereas the maximum for the lower-frequency EPU component changes from 0.108 to 0.208 and moves to a 2020Q2 shock. The corresponding component-specific maxima occur at 2015Q4 for traditional Chinese medicine, 2022Q2 for western medicine, 2013Q4 for medical service CPI, and 2008Q2 for pharmaceutical manufacturing PPI. Two features of the comparison stand out. First, the aggregate response remains centered on the 2020Q2 episode, while the maximum for the higher-frequency EPU component remains associated with the global financial crisis. Second, component-level peak timing changes more noticeably across sample endpoints, particularly for traditional Chinese medicine, medical services, and pharmaceutical manufacturing PPI. The two frequency-specific maxima also become nearly equal in the truncated sample. Both higher- and lower-frequency EPU components remain quantitatively relevant in the truncated sample. Supplementary Figure S1 and Supplementary Table S1 report the dates, horizons, and magnitudes for all seven surfaces and provide the complete comparison across sample endpoints.

Supplementary Table S3 and Supplementary Figure S2 assess sensitivity to the information set and factor count. Peak magnitudes and dates vary across specifications. For a 2020Q2 shock at an eight-quarter horizon, the aggregate responses are 0.130, 0.237, and −0.023 with two, three, and four factors, respectively. The frequency-specific peak ranking also changes across information sets. The sensitivity results therefore caution against treating any single ranking of response maxima as invariant across specifications.

Figure 7 provides complementary production-side evidence. Factory-gate pharmaceutical prices are shaped by a different set of margins from consumer-facing medical prices, including input costs, inventories, import conditions, and supply-chain adjustment. Procurement contracts, distribution margins, reimbursement arrangements, and administered prices then influence how upstream pressure is transmitted through the health-price system. Read together, Figures 6, 7 add two distinct dimensions to the component analysis. Medical service CPI captures a non-pharmaceutical consumer-side price margin, while pharmaceutical manufacturing PPI captures price adjustment at the production stage. Their different response profiles show that consumer-facing and upstream price pressures do not necessarily move in parallel. Examining both layers therefore provides a more complete picture of where EPU-related price adjustment appears within the medical system and how that adjustment differs across stages of price formation.

## 5 Discussion

The frequency-specific results indicate that medical-price responses differ across higher- and lower-frequency components of EPU and across historical periods. In the full sample, the posterior-mean maximum for the higher-frequency component is 0.297, compared with 0.108 for the lower-frequency component. Re-estimation through 2025Q4 narrows the corresponding maxima to 0.214 and 0.208, so their relative peak size depends on the sample endpoint. The evidence supports transmission from more than one EPU frequency band without implying that either frequency band is uniformly dominant. Higher-frequency uncertainty may capture shorter-cycle policy and financing disturbances, whereas lower-frequency uncertainty may reflect more persistent expectations surrounding procurement, reimbursement, and administered pricing. These channels operate within a health-price system shaped by contracts and public payment arrangements, rather than through instantaneous price adjustment alone. A single full-sample coefficient or constant-parameter VAR would miss variation across shock dates and response horizons. The wavelet decomposition identifies the frequency content of EPU, the SV-TVP-FAVAR traces medical-price responses over time, and the posterior summaries quantify uncertainty around those responses.

The aggregate response is elevated for a shock dated 2020Q2, reaching a posterior-mean maximum of 0.237 at an eight-quarter horizon with a central 68% credible interval of [0.063, 0.412]. At that horizon, its posterior band overlaps those at earlier comparison dates (Figure 8). The timing nevertheless coincides with the first phase of COVID-19, when health-service use, supply conditions, and policy implementation were undergoing unusually large adjustments. Studies cited in (43, 44) document the sharp contraction in health-service utilization during the early pandemic. Procurement, delivery, payment, and production decisions were also being made under heightened uncertainty. These conditions provide a plausible institutional context for the estimated response. Health-care prices are shaped by contracts, reimbursement schedules, procurement cycles, and administrative pricing rules rather than adjusted continuously. Such arrangements could allow an uncertainty shock to affect production and procurement decisions before becoming visible in consumer-facing prices, consistent with the eight-quarter horizon of the aggregate maximum.

Component heterogeneity is at least as important as historical timing. In the baseline full-sample specification, western medicine prices have the largest consumer-side posterior-mean maximum, 0.424, whereas traditional Chinese medicine prices peak at 0.197 and medical service CPI at 0.078. The aggregate maximum of 0.237 lies between the western medicine and traditional Chinese medicine responses, which is consistent with an index that combines stronger and weaker movements across its basket. The components are also exposed to different market conditions. Western medicine prices are more closely connected to manufacturing costs, imports, distribution networks, procurement rules, and regulatory expectations; traditional Chinese medicine prices have greater exposure to raw-material supply and seasonal market conditions; and medical service prices are shaped more directly by provider payment and administered pricing. These differences provide an economic interpretation for the baseline ranking of responses and show why aggregate health-care CPI alone cannot reveal where EPU-related price pressure is concentrated within the health system.

This component-level evidence moves the paper beyond expenditure-focused EPU-health research. Health-care expenditure combines prices, utilization, public budgets, insurance coverage, demographic demand, and changes in service intensity. A rise in expenditure can therefore reflect more care, broader coverage, emergency public support, or higher prices. Price indices isolate the price dimension more directly, and their components show where pressure appears within the medical system. That distinction matters for affordability and payment pressure because households, insurers, and public payers face the consequences of price changes even when utilization does not expand. This comparatively large western medicine response indicates that EPU-related price adjustment is particularly pronounced in a pharmaceutical pricing margin with direct relevance to the cost of care. The smaller responses in other components further show that medical-price adjustment is heterogeneous across the health-price system rather than a uniform movement in the aggregate index.

The policy implication follows from the same heterogeneity. Aggregate health-care CPI alone can conceal stronger movements in western medicine prices, while medicine-only monitoring would omit medical services and upstream pharmaceutical production. Neither the aggregate index nor one pharmaceutical component provides a complete account of the pricing environment. These differences support component-level monitoring, particularly in China, where procurement, reimbursement, distribution, producer costs, and provider pricing do not necessarily change together across the health sector. Monitoring these layers jointly makes it easier to distinguish a broad medical-price movement from pressure concentrated in a particular component. It also connects the empirical surfaces more directly to the institutional points at which policy uncertainty can enter production, payment, and final consumer prices.

Figures 6, 7 extend the analysis to medical services and upstream pharmaceutical production. The baseline medical-service response is comparatively modest, showing that the consumer-side relationship is not confined to medicine prices. Pharmaceutical manufacturing PPI displays a larger full-sample response and a distinct production-side profile. Factory-gate prices are shaped by input costs, inventories, import conditions, and supply-chain adjustment, whereas consumer indices also incorporate distribution, procurement, provider payment, and administered pricing. Pharmaceutical manufacturing PPI therefore adds an upstream dimension to the component-level evidence. This distinction also matters for public-health interpretation. Medical service CPI is closer to prices faced within the delivery system, whereas manufacturing PPI captures pressure before distribution and reimbursement arrangements intervene. Their different response profiles suggest that upstream cost pressure and consumer-facing adjustment do not necessarily coincide; the terminal 2026Q1 PPI maximum, however, is likely an endpoint effect. Monitoring only the final consumer index could therefore miss changes developing in pharmaceutical production, while monitoring only producer prices would overlook the payment and service-pricing conditions experienced by households, providers, and public payers.

The comparison with closely related studies further clarifies the paper’s contribution. Bai et al. (37) and Sui et al. (39) examine health-care or fiscal expenditure in China, outcomes that combine prices, quantities, and public spending decisions. The present paper instead follows medical-price indices and documents substantial variation across their components and response horizons. Cheng and Witvorapong (36) report negative responses of U. S. health-care inflation to health-care policy uncertainty. The difference in sign highlights the importance of both the uncertainty measure and the institutional setting in which health-care prices are formed. Their study focuses on health-policy-specific uncertainty in a different insurance, payment, and regulatory environment, whereas the present paper examines broad EPU in China. The Chinese evidence therefore extends the literature to a setting in which pharmaceutical production, centralized procurement, public payment expectations, and administered pricing play an important role in medical-price formation.

The posterior surfaces further show that historical timing and component composition are jointly important. Positive responses are concentrated at specific dates and horizons rather than spread uniformly across the sample. The baseline aggregate posterior mean is elevated around 2020Q2, and its eight-quarter band overlaps those at earlier dates. Western medicine prices display a broad elevated region after 2020. The component surfaces differ markedly in both magnitude and timing. These patterns indicate that EPU transmission through the health-price system is both episode-specific and component-specific. A common macroeconomic uncertainty shock can therefore be associated with different adjustment paths across medicines, medical services, and upstream pharmaceutical production, consistent with differences in procurement, production, reimbursement, and price-setting arrangements.

The posterior intervals provide complementary information on estimation uncertainty. At each reported posterior-mean maximum, the 16th–84th percentile interval lies above zero. Figure 8 separately shows how posterior uncertainty varies across shock dates at the eight-quarter horizon, while Supplementary Data S2 reports P16, the posterior mean, and P84 for every shock-date/horizon cell across the full response surfaces.

Several limitations also suggest directions for further research. The news-based EPU index captures broad economic policy uncertainty rather than uncertainty specific to health policy, reimbursement, or pharmaceutical regulation. Health-care CPI measures consumer-facing inflation but does not directly observe household out-of-pocket payments, procurement prices, reimbursement rates, provider costs, or changes in service quality. Pharmaceutical manufacturing PPI adds an upstream price measure, but the connection between factory-gate prices and consumer-facing medical prices depends on procurement, distribution, reimbursement, and provider-pricing arrangements that are not observed separately in the aggregate series. More granular data could therefore extend the analysis in several directions. Firm- and hospital-level information could link pharmaceutical input costs and producer prices to purchasing decisions and final prices; procurement and reimbursement data could identify how administrative rules shape the timing of pass-through; and household expenditure or claims data could connect measured medical inflation more directly to financial protection and access to care. Health-policy-specific uncertainty measures and cross-country applications would provide further evidence on how insurance design, market structure, pharmaceutical regulation, and health-system institutions shape the magnitude and timing of uncertainty-related medical inflation.

## 6 Policy implications

The findings have three policy implications. First, medical inflation monitoring should not rely only on aggregate health-care CPI or a single full-sample average. The posterior surfaces show that uncertainty-related price pressure is concentrated at particular dates, horizons, and components. Western medicine prices display the largest consumer-side maximum in the baseline, while traditional Chinese medicine and medical service prices follow different profiles. Regulators and health-system managers should therefore monitor the internal composition of the health-price basket when policy changes affect procurement, reimbursement, imports, or pharmaceutical supply conditions. A stable aggregate index can coexist with stronger movement inside one component, so component-level signals are useful for identifying where affordability and payment pressure may emerge.

Second, responses to both higher- and lower-frequency EPU components underscore the value of clear policy communication and implementation details beyond the immediate news cycle. Timely information about reimbursement rules, procurement programs, price regulation, and health-sector fiscal measures can reduce uncertainty surrounding financing, inventory, production, and pricing decisions. Predictable implementation schedules and payment conditions give firms and providers more time to adjust their plans, while clear transition arrangements can reduce abrupt changes in procurement and inventory behavior. The relevant policy objective is greater predictability in timing and implementation, enabling firms and providers to incorporate policy changes more effectively into financing, inventory, production, and pricing decisions.

Third, upstream and consumer-facing indicators provide complementary signals. Pharmaceutical manufacturing PPI adds a production-side view to the consumer price measures, while the CPI components indicate where price pressure is visible to households and payers. A practical monitoring framework can therefore combine aggregate health-care CPI, western and traditional medicine prices, medical service CPI, pharmaceutical producer prices, and relevant measures of policy uncertainty. Producer prices, input costs, inventories, and supply-chain conditions can help identify upstream pressure, while component-level consumer prices show how that pressure appears within the health-price basket. Because the response profiles differ across horizons, monitoring should also distinguish immediate movements from pressures that accumulate over several quarters. A short-run signal may call for attention to financing, inventories, or temporary supply disruption, whereas a delayed response may be more closely connected to procurement cycles, reimbursement schedules, and the implementation of administered pricing rules. This layered approach is more informative than treating aggregate medical inflation as a single contemporaneous outcome.

## 7 Conclusion

This study examined whether economic policy uncertainty enters China’s medical-price system, how responses vary across EPU frequency components and historical dates, and where they are most visible within the health-price basket. The baseline estimates show positive medical-inflation responses to both higher- and lower-frequency EPU components. The higher-frequency maximum exceeds the lower-frequency maximum in the full sample, whereas the two are nearly equal when the sample ends in 2025Q4. In the baseline three-factor model, the aggregate response is elevated around 2020Q2; its eight-quarter posterior band overlaps those at several earlier dates, and the estimated magnitude varies with factor count. Western medicine prices exhibit the largest consumer-side posterior-mean maximum in the baseline specification, while traditional Chinese medicine prices and medical service CPI respond more moderately. Pharmaceutical manufacturing PPI adds a production-side comparison, although its terminal 2026Q1 maximum is not reproduced in the truncated sample and is likely an endpoint effect. Re-estimation through 2025Q4 preserves the aggregate 2020Q2 episode while shifting several component peak dates. The evidence points to medical-price responses that are time-varying, multi-scale, and heterogeneous across components, with exact peak magnitudes and dates varying across specifications.

The main contribution is to connect EPU research with public-health and health-economics concerns about affordability, provider payment, procurement, and pharmaceutical pricing. By focusing on medical inflation rather than expenditure alone, the analysis separates price pressure from changes in utilization, insurance coverage, demographics, and public budgets. The component results further show why aggregate health-care CPI should be considered alongside medicine prices, service prices, and upstream producer prices. Methodologically, the combination of wavelet decomposition and an SV-TVP-FAVAR captures variation across timescales, shock dates, and response horizons, while the posterior summaries quantify uncertainty around the estimated responses. These findings support component-level monitoring and greater predictability in the implementation of procurement, reimbursement, and price-regulation policies.

The study uses a broad EPU measure rather than a health-policy-specific uncertainty index, and aggregate price indices cannot separately identify individual procurement, reimbursement, provider-cost, or producer-to-consumer pass-through mechanisms. Future research using health-policy-specific uncertainty measures and firm-, hospital-, procurement-, or reimbursement-level data can provide further evidence on these transmission mechanisms and assess their relevance in other health-system settings.

## Data Availability

The original monthly China EPU data based on the South China Morning Post (SCMP) are publicly available from the Economic Policy Uncertainty project: https://www.policyuncertainty.com/scmp_monthly.html. Health-price and macroeconomic series were obtained from the Wind database: https://www.wind.com.cn/. The processed quarterly dataset used in this study is available from the authors upon reasonable request, subject to data-provider restrictions.
