# Supplementary material for: Economic policy uncertainty and medical inflation in China: multi-scale linkages and time-varying responses
Source: Front Public Health. 2026 Oct 14;14:1910486. doi: 10.3389/fpubh.2026.1910486 (PMC13713414; doi:10.3389/fpubh.2026.1910486)
Supplement: Supplementary file 1 [file Supplementary_file_1.docx]

Supplementary Material

# 1 Supplementary Descriptive Statistics

Unit conventions: the China news-based EPU index is reported on its source scale (mean = 100). Aggregate health-care CPI inflation, traditional Chinese medicine price inflation, western medicine price inflation, medical service CPI, and pharmaceutical manufacturing PPI are year-on-year growth rates (%). Variables in Appendix Table A2 remain in the original source units reported by the underlying data sources.

**Appendix Table A1.** Descriptive statistics for core uncertainty and health-price variables

| **Variable** | **Unit** | **N** | **Mean** | **SD** | **Excess kurtosis** | **Skewness** | **Min** | **Max** |
| --- | --- | --- | --- | --- | --- | --- | --- | --- |
| China: News-Based EPU | Mean=100 | 89 | 277.211 | 245.062 | -0.098 | 1.012 | 43.794 | 1011.689 |
| China: CPI: Health Care: YoY | % | 89 | 1.944 | 1.58 | 1.141 | 0.916 | -1.0 | 6.913 |
| China: CPI: Health Care: Traditional Chinese Medicine: YoY | % | 89 | 3.856 | 3.994 | 0.377 | 0.395 | -4.3 | 14.467 |
| China: CPI: Health Care: Western Medicines: YoY | % | 89 | 0.594 | 2.586 | 0.378 | 0.392 | -5.567 | 6.3 |
| China: CPI: Health Care: Medical Services: YoY | % | 89 | 2.211 | 1.837 | 1.605 | 1.475 | 0.233 | 8.4 |
| China: PPI: Manufacture of Medicines: YoY | % | 89 | 0.662 | 1.837 | 0.437 | -0.088 | -4.567 | 5.113 |

**Note:** The sample covers quarterly observations from 2004Q1 to 2026Q1. All health-price variables are year-on-year growth rates. The China news-based EPU index is the SCMP-based series distributed through the Economic Policy Uncertainty project.

**Appendix Table A2.** Descriptive statistics for the full macroeconomic information set

| **Category** | **Variable** | **Unit** | **N** | **Mean** | **SD** | **Excess kurtosis** | **Skewness** | **Min** | **Max** |
| --- | --- | --- | --- | --- | --- | --- | --- | --- | --- |
| Output and sectoral GDP | China: GDP: Current Prices | 100 million yuan | 89 | 183435.364 | 101495.798 | -1.172 | 0.26 | 35107.8 | 387911.3 |
| Output and sectoral GDP | China: GDP: Current Prices: Primary Industry | 100 million yuan | 89 | 14061.576 | 8147.837 | -0.017 | 0.829 | 2615.6 | 35159.7 |
| Output and sectoral GDP | China: GDP: Current Prices: Secondary Industry | 100 million yuan | 89 | 72335.274 | 34974.541 | -1.139 | 0.122 | 15292.1 | 136803.3 |
| Output and sectoral GDP | China: GDP: Current Prices: Tertiary Industry | 100 million yuan | 89 | 97038.512 | 60542.474 | -1.223 | 0.328 | 16717.4 | 215948.3 |
| Output and sectoral GDP | China: GDP: Current Prices: Agriculture, Forestry, Animal Husbandry and Fishery | 100 million yuan | 89 | 14661.689 | 8566.028 | 0.045 | 0.851 | 2698.4 | 37258.0 |
| Output and sectoral GDP | China: GDP: Current Prices: Industry | 100 million yuan | 89 | 60634.472 | 28043.21 | -1.137 | 0.089 | 14093.6 | 111454.7 |
| Output and sectoral GDP | China: GDP: Current Prices: Construction | 100 million yuan | 89 | 12070.32 | 7549.158 | -0.975 | 0.411 | 1242.8 | 28003.9 |
| Output and sectoral GDP | China: GDP: Current Prices: Wholesale and Retail Trade | 100 million yuan | 89 | 17554.843 | 10787.886 | -1.077 | 0.339 | 2969.4 | 40255.1 |
| Output and sectoral GDP | China: GDP: Current Prices: Transport, Storage and Post | 100 million yuan | 89 | 8058.608 | 4137.139 | -1.013 | 0.39 | 2155.9 | 16706.3 |
| Output and sectoral GDP | China: GDP: Current Prices: Hotel and Catering Services | 100 million yuan | 89 | 3250.425 | 1762.065 | -0.472 | 0.609 | 896.7 | 7898.6 |
| Output and sectoral GDP | China: GDP: Current Prices: Finance | 100 million yuan | 89 | 13047.589 | 7955.067 | -1.39 | 0.07 | 1515.3 | 27224.6 |
| Output and sectoral GDP | China: GDP: Current Prices: Real Estate | 100 million yuan | 89 | 13121.394 | 7391.012 | -1.54 | -0.076 | 2004.1 | 24120.1 |
| Output and sectoral GDP | China: GDP: Current Prices: Other Sectors | 100 million yuan | 89 | 30857.906 | 17233.776 | -1.05 | 0.347 | 6536.7 | 65687.3 |
| Real activity and domestic demand | China: Industrial Added Value: Industrial Enterprises Above Designated Size: YoY (January-February Split) | % | 89 | 9.66 | 5.693 | 2.046 | 0.39 | -10.427 | 30.613 |
| Aggregate price indicators | China: CPI: YoY | % | 89 | 2.216 | 1.873 | 1.116 | 0.901 | -1.533 | 8.033 |
| Aggregate price indicators | China: PPI: YoY | % | 89 | 1.357 | 4.501 | -0.897 | 0.192 | -7.683 | 12.233 |
| External trade | China: Value of Imports and Exports | 100 million USD | 89 | 3305.961 | 1355.713 | -0.939 | -0.028 | 799.587 | 5640.028 |
| External trade | China: Exports | 100 million USD | 89 | 1837.729 | 798.112 | -0.882 | 0.127 | 385.737 | 3307.476 |
| External trade | China: Imports | 100 million USD | 89 | 1468.231 | 565.26 | -0.981 | -0.22 | 413.853 | 2381.889 |
| Real activity and domestic demand | China: FAI: YTD | 100 million yuan | 89 | 196510.543 | 168433.424 | -0.656 | 0.768 | 5172.755 | 597490.0 |
| Real activity and domestic demand | China: Total Retail Sales of Consumer Goods | 100 million yuan | 89 | 23139.312 | 12717.35 | -1.405 | 0.003 | 4139.533 | 45108.4 |
| Money, credit, and exchange rates | China: M0 | 100 million yuan | 89 | 64386.677 | 32822.71 | -0.335 | 0.616 | 19314.803 | 148219.273 |
| Money, credit, and exchange rates | China: M1 | 100 million yuan | 89 | 433863.253 | 293938.222 | 0.407 | 0.992 | 84392.633 | 1177380.777 |
| Money, credit, and exchange rates | China: M2 | 100 million yuan | 89 | 1438223.486 | 963251.18 | -0.918 | 0.504 | 227935.75 | 3500885.61 |
| Money, credit, and exchange rates | China: Loan Balance of Financial Institutions: RMB | 100 million yuan | 89 | 1080732.519 | 806719.913 | -0.842 | 0.66 | 164327.927 | 2782165.197 |
| Money, credit, and exchange rates | Central Parity Rate: USDCNY | CNY per USD | 89 | 6.919 | 0.608 | 0.058 | 0.895 | 6.118 | 8.277 |
| Money, credit, and exchange rates | Central Parity Rate: EUR/CNY | CNY per EUR | 89 | 8.477 | 1.151 | -0.782 | 0.683 | 6.778 | 10.881 |
| Money, credit, and exchange rates | Central Parity Rate: HKD/CNY | CNY per HKD | 89 | 0.889 | 0.078 | 0.118 | 0.943 | 0.789 | 1.064 |
| Financial markets | SH and SZ Stock Markets: A and B Shares: Total Market Value | 100 million yuan | 89 | 433244.702 | 294662.879 | -0.956 | 0.41 | 31632.873 | 1147384.985 |
| Financial markets | SH and SZ Stock Markets: A and B Shares: Market Value of Shares in Circulation | 100 million yuan | 89 | 344943.275 | 272213.796 | -0.794 | 0.515 | 10217.457 | 1039891.615 |
| Financial markets | SH and SZ Stock Markets: A and B shares: Number of Listed Companies | count | 89 | 2984.013 | 1314.951 | -1.207 | 0.376 | 1295.0 | 5194.5 |
| Financial markets | SSE Conglomerates Index | index points | 89 | 2831.062 | 817.83 | 0.622 | -0.006 | 1117.633 | 5367.816 |
| Financial markets | CSI 300 Index | index points | 89 | 3206.475 | 1133.311 | -0.436 | -0.39 | 888.798 | 5354.578 |
| Financial markets | Hang Seng China AH Premium Index | index points | 81 | 125.517 | 18.016 | -0.216 | 0.064 | 91.9 | 180.27 |
| Financial markets | SZSE Composite Index | index points | 89 | 1422.617 | 655.307 | -1.03 | -0.141 | 270.159 | 2669.145 |
| Financial markets | SSE T-Bond Index: Closing | index points | 89 | 152.528 | 37.548 | -0.961 | 0.362 | 94.095 | 225.481 |
| Fiscal conditions and health budget | China: General Public Budget Revenue (January-February Combined) | 100 million yuan | 89 | 12153.86 | 7366.374 | 0.527 | 0.819 | 1980.233 | 31170.5 |
| Fiscal conditions and health budget | China: Tax Revenue: Company Income Tax (January-February Combined) | 100 million yuan | 89 | 2297.538 | 1627.618 | -0.385 | 0.856 | 278.55 | 5932.0 |
| Fiscal conditions and health budget | China: Tax Revenue: Individual Income Tax (January-February Combined) | 100 million yuan | 89 | 787.686 | 555.171 | 1.163 | 1.163 | 130.865 | 2509.0 |
| Fiscal conditions and health budget | China: General Public Budget Revenue: Budget: Total | 100 million yuan | 89 | 129588.77 | 68384.449 | -1.426 | -0.204 | 21228.917 | 223950.0 |
| Fiscal conditions and health budget | China: General Public Budget Expenditure: Budget: Health and Family Planning | 100 million yuan | 89 | 10978.392 | 8121.831 | -1.188 | 0.314 | 727.732 | 26640.919 |
| Business-cycle indicators | China: Macro-economic Climate Index: Coincident Index | index | 89 | 99.721 | 3.732 | 4.115 | 0.472 | 87.076 | 115.65 |
| Business-cycle indicators | China: Macro-economic Climate Index: Leading Index | index | 89 | 100.763 | 2.652 | 0.633 | -0.583 | 92.633 | 105.533 |
| Business-cycle indicators | China: Macro-economic Climate Index: Lagging Index | index | 89 | 97.757 | 2.87 | 0.191 | -0.288 | 90.033 | 103.777 |
| Business-cycle indicators | China: Business Climate Index | index | 89 | 122.275 | 12.013 | -0.479 | -0.232 | 88.217 | 146.0 |
| Household income and inequality | China: per Capita Disposable Income of Urban Households: YTD | yuan | 89 | 19749.217 | 13312.23 | 0.125 | 0.948 | 2638.8 | 56502.0 |
| Household income and inequality | China: per Capita Consumption Expenditure of Rural Households | yuan | 89 | 9324.509 | 5777.224 | -1.146 | 0.413 | 2001.015 | 20605.082 |
| Household income and inequality | China: Gini Coefficient of Disposable Income of Households | coefficient | 89 | 0.473 | 0.009 | -0.989 | 0.692 | 0.462 | 0.491 |
| Demographics | China: Total Population | 10,000 persons | 89 | 136894.369 | 3975.841 | -1.336 | -0.41 | 129416.831 | 141260.0 |
| Demographics | China: Total Population: Male | 10,000 persons | 89 | 70182.131 | 1841.134 | -1.34 | -0.383 | 66660.752 | 72357.0 |
| Demographics | China: Total Population: Female | 10,000 persons | 89 | 66712.225 | 2137.372 | -1.327 | -0.429 | 62756.077 | 68969.0 |
| Demographics | China: Total Population: Aged 15 to 64 | 10,000 persons | 89 | 97870.024 | 2679.356 | -0.642 | -0.462 | 91276.508 | 101041.0 |
| Demographics | China: Total Population: 65Y Old and Above | 10,000 persons | 89 | 15034.94 | 4212.822 | -1.176 | 0.462 | 9732.989 | 23229.415 |
| Input costs and energy | Spot Price: Crude Oil: West Texas Intermediate (WTI) | USD/barrel | 89 | 69.369 | 20.875 | -0.473 | 0.352 | 29.200 | 124.230 |
| Health-sector demand | China: Retail Sales: Traditional Chinese and Western Medicines: Units Above Designated Size | 100 million yuan | 89 | 424.680 | 222.640 | -1.154 | -0.205 | 76.567 | 877.900 |
| Money, credit, and exchange rates | China: Aggregate Financing to the Real Economy (AFRE): Flow | 100 million yuan | 89 | 16377.690 | 11100.147 | 1.290 | 1.076 | 1306.000 | 50612.667 |

Note: The 56 series are listed in original source units before stationarity transformation and standardization. Kurtosis is excess kurtosis.

# 2 Supplementary Robustness and Posterior Uncertainty

To assess sensitivity to the most recent observation, all seven SV-TVP-FAVAR specifications were re-estimated on a sample ending in 2025Q4. Supplementary Figure S1 reports the corresponding posterior-mean surfaces, and Supplementary Table S1 compares their maxima with the full-sample posterior-mean maxima. Supplementary Table S2 reports the 16th percentile (P16), posterior mean, and 84th percentile (P84) at each full-sample maximum. Complete cell-level posterior summaries for all shock dates and horizons are provided in Supplementary Data S2. Supplementary Table S3 compares response-surface maxima across the 53-variable specification and the 56-variable specifications with two, three, and four factors. Supplementary Figure S2 displays the corresponding selected eight-quarter response profiles.

**Supplementary Figure S1. Posterior-mean response surfaces from the sample truncated at 2025Q4**

*Supplementary Figure S1 continues on the next page.*

**Supplementary Figure S1 (continued)**

*Note: Each panel reports the posterior-mean response to a one-standard-deviation EPU shock over horizons of 1-15 quarters. The horizontal axis records the shock date, the vertical axis records the response horizon, and color represents the impulse-response magnitude. The estimation sample ends in 2025Q4. In panels A and B, the frequency labels refer to EPU shock components; the medical-price outcome remains unfiltered.*

**Supplementary Table S1. Full-sample and 2025Q4-truncated peak responses**

| **Response specification** | **Full peak** | **Shock date** | **h** | **Truncated peak** | **Shock date** | **h** |
| --- | --- | --- | --- | --- | --- | --- |
| Higher-frequency EPU component | 0.297 | 2008Q3 | 6 | 0.214 | 2008Q3 | 5 |
| Lower-frequency EPU component | 0.108 | 2013Q2 | 7 | 0.208 | 2020Q2 | 7 |
| Aggregate health-care CPI | 0.237 | 2020Q2 | 8 | 0.376 | 2020Q2 | 11 |
| Traditional Chinese medicine prices | 0.197 | 2021Q2 | 5 | 0.316 | 2015Q4 | 9 |
| Western medicine prices | 0.424 | 2020Q3 | 6 | 0.246 | 2022Q2 | 5 |
| Medical service CPI | 0.078 | 2014Q1 | 8 | 0.176 | 2013Q4 | 7 |
| Pharmaceutical manufacturing PPI | 0.338 | 2026Q1 | 6 | 0.330 | 2008Q2 | 5 |

*Note: Full-sample and truncated-sample columns report posterior-mean maxima from estimations ending in 2026Q1 and 2025Q4, respectively. Peak dates and horizons are descriptive features of the response surfaces. Posterior uncertainty for the full-sample maxima is reported separately in Supplementary Table S2.*

Supplementary Table S2. Posterior uncertainty at the full-sample posterior-mean maxima

| **Response specification** | **Shock date** | **Horizon (quarters)** | **Posterior mean** | **P16** | **P84** | **Central 68% credible interval** |
| --- | --- | --- | --- | --- | --- | --- |
| Higher-frequency EPU component | 2008Q3 | 6 | 0.297 | 0.122 | 0.472 | [0.122, 0.472] |
| Lower-frequency EPU component | 2013Q2 | 7 | 0.108 | 0.035 | 0.181 | [0.035, 0.181] |
| Aggregate health-care CPI | 2020Q2 | 8 | 0.237 | 0.063 | 0.412 | [0.063, 0.412] |
| Traditional Chinese medicine prices | 2021Q2 | 5 | 0.197 | 0.126 | 0.268 | [0.126, 0.268] |
| Western medicine prices | 2020Q3 | 6 | 0.424 | 0.231 | 0.618 | [0.231, 0.618] |
| Medical service CPI | 2014Q1 | 8 | 0.078 | 0.046 | 0.109 | [0.046, 0.109] |
| Pharmaceutical manufacturing PPI | 2026Q1 | 6 | 0.338 | 0.115 | 0.561 | [0.115, 0.561] |

Note: P16 and P84 are the lower and upper bounds of the central 68% credible interval at each reported full-sample posterior-mean maximum. Supplementary Data S2 reports P16, the posterior mean, and P84 for every shock-date × horizon cell in Figures 1-7.

Supplementary Table S3. Information-set and factor-count sensitivity of response maxima

| Response specification | 53 variables, K = 3 | 56 variables, K = 3 | 56 variables, K = 2 | 56 variables, K = 4 |
| --- | --- | --- | --- | --- |
| Higher-frequency EPU component | 0.142 (2010Q4, h=2) | 0.297 (2008Q3, h=6) | 0.208 (2020Q1, h=6) | 0.295 (2005Q3, h=4) |
| Lower-frequency EPU component | 0.357 (2020Q2, h=5) | 0.108 (2013Q2, h=7) | 0.150 (2025Q3, h=4) | 0.211 (2014Q4, h=6) |
| Aggregate health-care CPI | 0.265 (2020Q2, h=8) | 0.237 (2020Q2, h=8) | 0.241 (2010Q1, h=8) | 0.101 (2014Q2, h=2) |
| Traditional Chinese medicine | 0.603 (2022Q1, h=15) | 0.197 (2021Q2, h=5) | 0.211 (2023Q3, h=5) | 0.273 (2021Q2, h=8) |
| Western medicine | 0.649 (2024Q4, h=8) | 0.424 (2020Q3, h=6) | 0.512 (2015Q4, h=15) | 0.575 (2020Q2, h=4) |
| Medical service CPI | 0.178 (2025Q4, h=4) | 0.078 (2014Q1, h=8) | 0.073 (2014Q2, h=4) | 0.241 (2023Q2, h=4) |
| Pharmaceutical manufacturing PPI | 0.194 (2007Q1, h=5) | 0.338 (2026Q1, h=6) | 0.337 (2022Q1, h=4) | 0.359 (2025Q3, h=2) |

Note: Each entry reports the posterior-mean maximum within horizons 1–15, together with the corresponding shock date and response horizon. The 53-variable and 56-variable estimates were produced in separate estimation rounds, so their comparison is descriptive. The K = 2 and K = 4 variants use the 56-variable information set.

Supplementary Figure S2. Eight-quarter posterior-mean response profiles for the higher-frequency EPU component, the lower-frequency EPU component, and aggregate health-care CPI under the 53-variable three-factor specification and the 56-variable specifications with two, three, and four factors. The 53-variable and 56-variable estimates were produced in separate estimation rounds, so the information-set comparison is descriptive.
